# Multi-scale characterisation of a ferroelectric polymer reveals the emergence of a morphological phase transition driven by temperature

**DOI:** 10.1038/s41467-020-20407-6

**Published:** 2021-01-08

**Authors:** Jonas Hafner, Simone Benaglia, Filipe Richheimer, Marco Teuschel, Franz J. Maier, Artner Werner, Sebastian Wood, Daniel Platz, Michael Schneider, Klaudia Hradil, Fernando A. Castro, Ricardo Garcia, Ulrich Schmid

**Affiliations:** 1 Institute of Sensor and Actuator Systems, TU Wien, Gusshausstrasse 27-29, 1040 Vienna, Austria; 2 Instituto de Ciencia de Materiales de Madrid, CSIC, Sor Juana Inés de la Cruz 3, 28049 Madrid, Spain; 3 National Physical Laboratory, Hampton Road, Teddington, TW11 0LW UK; 4 X-ray Centre, TU Wien, Getreidemarkt 9, 1060 Vienna, Austria

**Keywords:** Ferroelectrics and multiferroics, Phase transitions and critical phenomena, Polymers, Mechanical properties

## Abstract

Ferroelectric materials exhibit a phase transition to a paraelectric state driven by temperature - called the Curie transition. In conventional ferroelectrics, the Curie transition is caused by a change in crystal symmetry, while the material itself remains a continuous three-dimensional solid crystal. However, ferroelectric polymers behave differently. Polymeric materials are typically of semi-crystalline nature, meaning that they are an intermixture of crystalline and amorphous regions. Here, we demonstrate that the semi-crystalline morphology of the ferroelectric copolymer of vinylidene fluoride and trifluoroethylene (P(VDF-TrFE)) strongly affects its Curie transition, as not only a change in crystal symmetry but also in morphology occurs. We demonstrate, by high-resolution nanomechanical measurements, that the semi-crystalline microstructure in the paraelectric state is formed by crystalline domains embedded into a softer amorphous phase. Using in situ X-ray diffraction measurements, we show that the local electromechanical response of the crystalline domains is counterbalanced by the amorphous phase, effectively masking its macroscopic effect. Our quantitative multi-scale characterisations unite the nano- and macroscopic material properties of the ferroelectric polymer P(VDF-TrFE) through its semi-crystalline nature.

## Abstract (web summary)

Ferroelectric polymeric materials possess intermixture of crystalline and amorphous regions with complex Curie transition. Here, the authors demonstrate that the semi-crystalline morphology of the ferroelectric copolymer of P(VDF-TrFE) strongly affects its Curie transition.

## Introduction

Ferroelectrics are polar materials in which the spontaneously generated electric polarisation can be reversed by an external electric field. Consequently, they exhibit two stable polarised states in the absence of an electric field – called the remnant polarisation. Switching between opposite polarities can be induced by a sufficiently high electric field – called the coercive field. The resulting hysteretic response between polarisation and electric field is characteristic for ferroelectrics^1–8^.

A concomitant interconversion between electrical and mechanical energy is observed for all ferroelectrics, originating from piezoelectricity in the material^9–14^. Depending on the direction of the energy conversion, a distinction is made between the direct and inverse piezoelectric effect. Both are thermodynamically equivalent and reversible. For the direct effect, electrical charge is generated in response to an external mechanical stress. Naturally, the opposite case is observed for the inverse effect. The application of an electric field to a piezoelectric material induces a change in its dimensions, typically leading to a mechanical strain of a few parts-per-thousand in thin films. For instance, a micrometre-thin piezoelectric would change its thickness by a few nanometres^8^.

As expected, ferroelectricity is not observable at all temperatures. Under the influence of rising temperature, the spontaneous polarity becomes thermodynamically unstable and a phase transition to a non-polar paraelectric state occurs^5–8^. This phase transition is known as the Curie transition and the critical temperature marking this transition is called the Curie temperature. As the increase of temperature causes formation of a more disordered phase, ferroelectricity is typically found below the Curie transition. Above the Curie transition in the paraelectric state, the characteristic hysteretic response between polarisation and electric field disappears. Consequently, ferroelectrics do not show remnant polarisation in the paraelectric state.

Conventional ferroelectrics such as barium titanate (BaTiO_3_) are continuous three-dimensional (3D) solid crystals with strong ionic/covalent bonds^8^. For such ferroelectrics, the Curie transition is caused by a change in crystal symmetry from a non-centrosymmetric to centrosymmetric crystal structure^15,16^. As a result, the ferroelectric crystal loses the ability to spontaneously generate polarisation and, thus, forms a non-polar paraelectric state. Despite the fact that the ferroelectric changes its crystal structure, the material itself remains a 3D solid crystal.

In contrast, ferroelectric polymers possess a much more complex morphology. They typically do not only consist of a pure crystalline structure but rather are an intermixture of crystalline (ordered) and amorphous (disordered) regions – called semi-crystalline morphology. Therefore, a structural hierarchy from the nano- to macroscale is typically found in polymeric materials. For instance, the ferroelectric polymer poly(vinylidene fluoride) (PVDF) crystallises in the form of spherulites, consisting of stacks of crystalline lamellae, which grow outward from a central nucleation point^2,17,18^. In the gaps between the lamellae, amorphous interlamellar regions are formed. The spherulites have a diameter of several microns, whereas the thickness of the lamellae is only few tens of nanometres. Naturally, the different structural features interact with each other at all hierarchical levels and it is also this interaction that renders the macroscopic material properties of ferroelectric polymers. Recently, it has been reported that the electromechanical response of PVDF and its copolymer of vinylidene fluoride and trifluoroethylene (P(VDF-TrFE)) relies on an electromechanical coupling between the intermixed crystalline lamellae and amorphous interlamellar regions^17^. Consequently, it is essential to study not only the macroscopic but also the nano- and microscopic material properties, to fully understand the macroscopic material properties of ferroelectric polymers.

In ferroelectric polymers, such as P(VDF-TrFE), a phase transition from a ferro- to paraelectric state driven by temperature occurs^19–30^. The Curie transition of ferroelectric polymers is also associated with a change in crystal symmetry within the crystalline lamellae. However, the question arises to what extent the semi-crystalline morphology and, in particular, the amorphous regions affect the Curie transition of ferroelectric polymers. For instance, it has been reported that a significant increase of the amorphous proportion is observed upon heating^19,30,31^. However, an increase of the amorphous proportion can only occur if the morphology of the polymer changes. In addition, an anomalous behaviour of the dielectric properties of P(VDF-TrFE) have been measured around the Curie transition^21,32–34^. For instance, the Curie transition shows a characteristic similar to that of relaxor ferroelectrics^21^. Apparently, a change in the crystal structure of the semi-crystalline morphology alone cannot be accounted for the Curie transition characteristic of ferroelectric polymers. Therefore, other effects that may originate from the semi-crystalline microstructure should be considered.

Here we demonstrate that, indeed, the semi-crystalline microstructure plays a decisive role for a full understanding of the macroscopic material properties of P(VDF-TrFE) at different temperatures. In particular, we show that when the ferroelectric polymer passes the Curie transition, not only a change in crystal symmetry but also in morphology is observed, which in turn significantly affect the macroscopic mechanical and electromechanical properties. To address the impact of the semi-crystalline morphology on the Curie transition demands multi-scale characterisations (from the nano- to macroscale) of the mechanical and electromechanical properties of P(VDF-TrFE) below and above the Curie transition. To that end, we have performed bimodal atomic force microscopy (AFM) measurements to generate high-resolution morphological and nanomechanical maps of P(VDF-TrFE). These measurements show unambiguously the emergence of a morphological phase transition driven by temperature. Above the Curie transition, P(VDF-TrFE) exhibits a morphology that leads to completely different micro- and macroscopic material properties. As the polymer exhibits crystalline domains for both the ferro- and paraelectric state, we have performed in situ grazing incidence X-ray diffraction (XRD) measurements to obtain the microscopic response of P(VDF-TrFE) during electrical stimulation^17^. Those measurements reveal a strong electromechanical strain (~1%) above the Curie transition in the crystalline domains with an average size of 100 nm. At larger scales, such as for 1 μm-thin films or thicker, the above response is not observed. Obviously, the microscopic electromechanical deformation vanishes across the bulk material due to a change in the microstructure of P(VDF-TrFE). The disappearance of the microscopic strain across the bulk material is explained by the increase and mechanical properties of the amorphous regions. The macroscopic electromechanical response of the bulk material has been measured as a change in film thickness on application of an electric field by in situ cantilever-based deflection measurements using a standard AFM^35,36^. The macroscopic mechanical properties of P(VDF-TrFE) have been measured using a dynamic mechanical analysis (DMA). Overall, our quantitative multi-scale characterisations unite the nano- and macroscopic material properties of the ferroelectric polymer P(VDF-TrFE) through its semi-crystalline nature.

## Results

### Nano- and microstructure of P(VDF-TrFE)

**Fig. 1:**
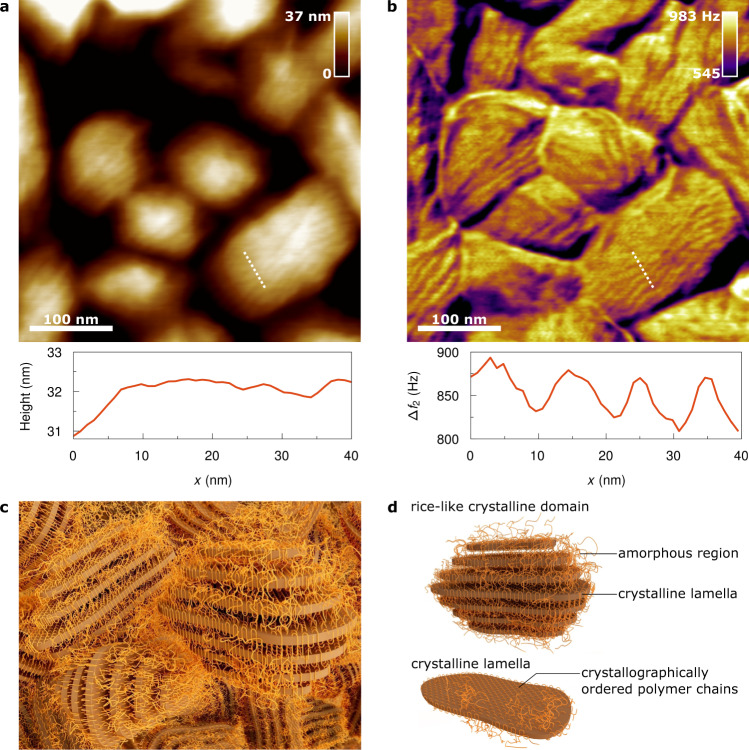
Nano- and microstructure of the semi-crystalline polymer P(VDF-TrFE). **a**, **b** High-resolution bimodal AFM images of P(VDF-TrFE), showing the true topography (height) and the frequency shift of the second mode (Δ*f*_2_). The profile of a cross-section through the lamellar structure of the crystalline domains is shown under each image, respectively. The bimodal AFM measurements displayed were performed at 300 K. **c** 3D illustration of the morphology of the semi-crystalline polymer P(VDF-TrFE), showing the ‘intermixture’ of crystalline and amorphous regions. **d** 3D illustration of a rice-like crystalline domain and a crystallographically ordered lamella of P(VDF-TrFE).

Fig. 1a, b show the composite nano- and microstructure of P(VDF-TrFE) given by high-resolution bimodal AFM. The polymer crystallises in the form of rice-like domains, consisting of stacks of lamellae^17,18,37–39^. The polymer chains are ordered in crystalline formations within the platelet-like lamellae, while in the gaps between lamellae the polymer chains adopt a disordered conformation, which produces the amorphous interlamellar regions. The alternating stacks of crystalline lamellae and amorphous interlamellar regions are clearly visible in Fig. 1b. The lamellae were found to have a thickness of about 5–8 nm and a distance between each other within one single domain of ~10 nm, which agrees with results reported previously^17–20,38^. A single polymer chain folds back and forth multiple times through the same lamellae or across different lamellae. As a consequence, the crystalline and amorphous regions are strongly intertwined within a single domain. Between the rice-like domains, the polymer chains form an amorphous region. For a better understanding of this, a 3D illustration of the nano- and microstructure is shown in Fig. 1c, d. Details of the synthesis of the polymer P(VDF-TrFE) used and its microstructure are presented in Supplementary Section 1, 2 and 3.

At ambient temperature and pressure, P(VDF-TrFE) is present in the low-temperature ferroelectric phase^17,18,20,40,41^. In this highly polar phase, the polymer chains preferentially possess the all trans conformation. In the literature, it is also often referred to as the β-phase due to its similarity to the β-phase of the homopolymer PVDF (see Supplementary Section 4 for details). All dipole moments of the polymer chains are aligned in the same direction, leading to a macroscopically observable spontaneous polarisation in the bulk material.

### Ferroelectric characteristics of P(VDF-TrFE)

**Fig. 2:**
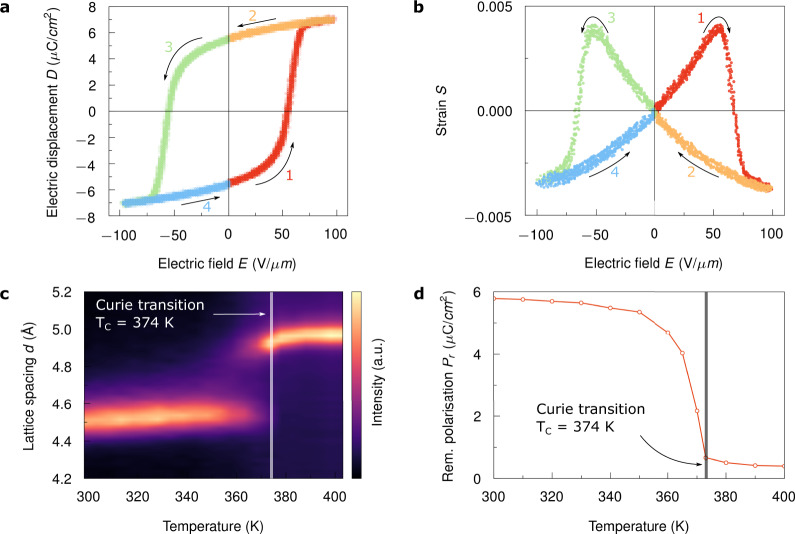
Ferroelectric characteristics of P(VDF-TrFE). **a** Electric displacement *D* as a function of the applied electric field *E*, resulting in ferroelectric *D*–*E* hysteresis loops. **b** Macroscopic strain *S* as a function of the field *E* yields the typically shaped butterfly curve. Both *D*–*E* and *S*–*E* were measured in the ferroelectric state at 300 K. Here, the capacitor was driven with a 10 Hz triangular waveform. **c** Specular XRD measurements, showing the lattice spacing *d* as a function of temperature (crystalline phase transition). **d** Remnant polarisation *P*_r_ as a function of temperature. The solid line through the measured values is a guide for the eye.

Consequently, the polymer exhibits ferroelectric behaviour. The hysteretic response of electric displacement *D* with applied electric field *E* is shown in Fig. 2a. A remnant polarisation *P*_r_ = 5.8 μC cm^−2^ and a coercive field of *E*_c_ = 55 V μm^−1^ are extracted. The concomitant macroscopic electromechanical deformation is displayed in Fig. 2b. The corresponding mechanical strain *S* = Δ*l*/*l*_0_ is calculated as the relative change in the thickness of the polymer film; here, Δ*l* is the change in the thickness of the polymer film from its zero-field value *l*_0_. Due to the distinctive shape of the *S*–*E* behaviour, it is called butterfly curve. For a better understanding of the correlation between the electrical and electromechanical performance of P(VDF-TrFE), the *D*–*E* hysteresis loop and the *S*–*E* butterfly curve are divided into four coloured segments, with the electric field sequence indicated by the arrows and numbers. Starting from a negatively polarised film, the polarisation switches to a positive orientation with increasing electric field at the coercive field *E*_c_ and finally starts to saturate at higher voltages (see segment 1 in Fig. 2a). A subsequent decrease of the voltage results in a slight reduction of the saturated polarisation towards the remnant polarisation *P*_r_ at zero-field (see segment 2 in Fig. 2a). Simultaneously, the mechanical strain increases from its initial zero value to a positive maximum at *E*_c_ and suddenly reverses sign after polarisation switching is completed (see segment 1 in Fig. 2b). Subsequently, electric field and polarisation are aligned and the strain saturates to a negative maximum. As the electric field starts to decrease again the strain decreases as well, until it vanishes as the polarisation reaches its remnant polarisation (see segment 2 in Fig. 2b). Segments 3 and 4 show the inverse process of segments 1 and 2. The *D*–*E* hysteresis loop and the concomitant electromechanical deformation were measured simultaneously using in situ cantilever-based deflection measurements^29,35,36^ in combination with a Sawyer–Tower setup. For that purpose, micromachined P(VDF-TrFE) capacitors were used. Details of the fabrication process and the measurements performed are presented in Supplementary Section 2, 4 and 8.

P(VDF-TrFE) contracts when an electric field is applied in the direction of its polarisation, resulting in a negative piezoelectric coefficient^17,42,43^. In general, it has been shown that the mechanical strain *S* in ferroelectric polymers varies as a square of the electric displacement *D* according to

1 $$S = Q_{33}D^2 = Q_{33}\left( {\varepsilon _0\varepsilon _{\mathrm{r}}E + P_{\mathrm{s}}} \right)^2 \, = Q_{33}\varepsilon _0^2\varepsilon _{\mathrm{r}}^2E^2 + 2Q_{33}\varepsilon _0\varepsilon _{\mathrm{r}}P_{\mathrm{s}}E + Q_{33}P_{\mathrm{s}}^2,$$

where *Q*_33_ is the longitudinal electrostriction coefficient, *ε* = *ε*_0_*ε*_r_ is the dielectric permittivity and *P*_s_ the spontaneous polarisation^42,43^. The first term, $Q_{33}\varepsilon _0^2\varepsilon _{\mathrm{r}}^2E^2$, on the right-hand side of Eq. (1) corresponds to the pure electrostrictive effect. The second term, $2Q_{33}\varepsilon _0\varepsilon _{\mathrm{r}}P_{\mathrm{s}}E$, is referred to as the linear longitudinal piezoelectric effect with the piezoelectric coefficient $d_{33} = 2Q_{33}\varepsilon _0\varepsilon _{\mathrm{r}}P_{\mathrm{r}}$. The last term, $Q_{33}P_{\mathrm{s}}^2$, represents the spontaneous strain. The origin of piezoelectricity was attributed to electrostriction biased by spontaneous polarisation^17,42,43^. As a negative *Q*_33_ is extracted, consequently, *d*_33_ is negative as well. At a temperature of 300 K, we extracted $Q_{33} = - 1.7\,{\mathrm{m}}^4{\mathrm{C}}^{ - 2}$ and $d_{33} = - 26.2\,{\mathrm{pm}}\,{\mathrm{V}}^{ - 1}$. To corroborate the extracted *d*_33_ via nonlinear fitting of the *S*–*E* characteristic at high fields according to Eq. (1), we additionally measured *d*_33_ at low fields. A *d*_33_ = −27.7 pm V^−1^ was extracted via linear fitting, which agrees with the other results. It should be noted that the model according to Eq. (1) has been extended to consider also the electromechanical coupling between the interlamellar amorphous and lamellar crystalline regions in the rice-like crystalline domains of P(VDF-TrFE)^17^. However, Eq. (1) is sufficient for the determination of the total piezoelectric response. Supplementary Section 4 provides a comprehensive study on the ferroelectric characteristics of P(VDF-TrFE).

As the temperature increases, P(VDF-TrFE) performs a crystalline phase transition from the polar low-temperature ferroelectric phase to the non-polar high-temperature paraelectric phase, marking the Curie transition at a temperature of *T*_C_ = 374 K (see Fig. 2c)^19–30^. The all trans sequences gradually transform into a mixture of gauche bonds above *T*_C_ (see Supplementary Section 4 for details). As expected, P(VDF-TrFE) exhibits a significant drop of the remnant polarisation at *T*_C_ (see Fig. 2d). However, the remnant polarisation does not vanish completely^32,33^. At a temperature of 395 K, which is more than 20 K above the Curie temperature, a remnant polarisation *P*_r_ ≈ 0.4 μC cm^−2^ is still observable. *P*_r_ as a function of temperature was measured using a Sawyer–Tower setup (see Supplementary Section 4). For that purpose, an electric field of 100 V μm^−1^ was applied to the film. This field is sufficient for the nucleation and growth of ferroelectric domains in the high-temperature paraelectric state. However, it should be noted that at high temperatures and under high-field poling, ferroelectric domains form in situ, meaning that they are short-lived and largely disappear after removing the poling electric field^32,33,44–47^. We will see that this ferroelectric-like behaviour is also observable in the electrostrain characteristic of the crystalline domains above the Curie transition. In addition, we refer to Supplementary Section 4 for further information about field-induced phase transitions observed in the high-temperature paraelectric state of P(VDF-TrFE).

### Morphological change in the microstructure of P(VDF-TrFE) above the Curie temperature *T*_C_

To get an insight into the morphological and nanomechanical properties of the semi-crystalline microstructure of P(VDF-TrFE) for both the ferro- and paraelectric state, we have performed high-resolution nanomechanical measurements (Young’s modulus maps) with a bimodal AFM at two temperatures (300 K and 395 K)^48–51^. A crucial part in performing these measurements is the control of the sample temperature. A custom-built heating system, ensuring a high stability of the sample temperature, was specifically built for this purpose, as further described in Supplementary Section 5. Details of the bimodal AFM measurements performed are described in Supplementary Section 6.

**Fig. 3:**
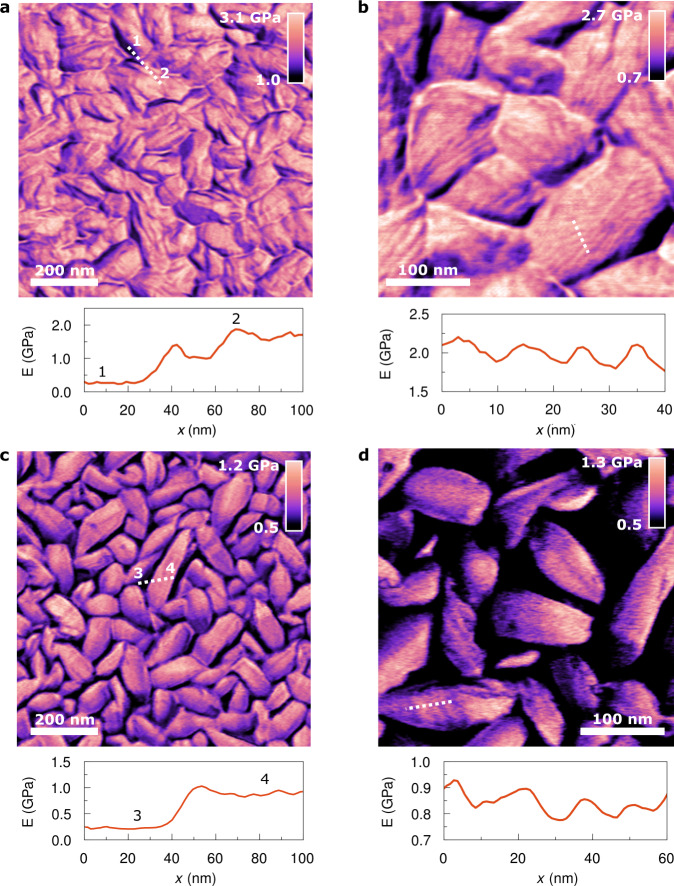
Young’s modulus maps of P(VDF-TrFE) below and above the Curie transition. **a**, **b** Spatially resolved nanomechanical maps of P(VDF-TrFE) for the low-temperature ferroelectric state at 300 K. **c**, **d** Nanomechanical maps of P(VDF-TrFE) for the high-temperature paraelectric state at 395 K. The bottom panels show the cross-sections along the lines traced in the images. Lamellar structures are observed below and above the Curie transition (*T*_C_ = 374 K). The nanomechanical maps have been generated by bimodal AFM.

The Young’s modulus maps of P(VDF-TrFE) measured for the low-temperature ferroelectric state at 300 K are shown in Fig. 3a, b. Below the Curie transition, the crystalline domains are densely packed analogously to a polycrystalline material with occasional amorphous regions at the grain boundaries. In Fig. 3b, the lamellar structure of the crystalline domains is observed as a subtle difference in the Young’s modulus, having a thickness of around 10 nm. In addition, we note that the orientation of the crystalline domains is similar to other spin-coated P(VDF-TrFE) thin films reported in the literature^52–54^. The amorphous regions outside of the crystalline domains form closed islands. These are clearly visible by the difference in Young’s modulus. A cross-section in Fig. 3a shows the contrast between the two material phases. The amorphous regions (1 in Fig. 3a) possess a Young’s modulus in the range of 0.3 ± 0.1 GPa, which is much lower than that of the crystalline domains (2 in Fig. 3a) with 1.7 ± 0.5 GPa.

In contrast, the high-temperature paraelectric state exhibits a different microstructure. Above the Curie transition at 395 K, the crystalline domains are no longer densely packed but separated from each other by amorphous regions (see Fig. 3b, c). The amorphous regions form a soft matrix in which the stiffer crystalline domains are embedded. A cross-section in Fig. 3c shows the difference between the crystalline domains and the amorphous regions. From an amorphous region (3 in Fig. 3c), the Young’s modulus significantly rises to the value of the crystalline domain (4 in Fig. 3c). Fig. 3d shows that above the Curie transition the polymer is formed by crystalline domains made of stacks of lamellae and amorphous interlamellar regions. The thickness of the lamellae is with around 18 nm significantly increased from the low-temperature to high-temperature phase. In the literature, a similar increase in the thickness of the lamellae has been observed, corroborating the results of Fig. 3d^19^.

A striking feature of the microstructure of P(VDF-TrFE) is the substantial change in the proportion of amorphous region, which occurs at the Curie transition. The proportion of outer amorphous regions is 2.5% ± 0.9% for the low-temperature ferroelectric state, while this is significantly higher for the high-temperature paraelectric state with 37.7% ± 3.8%. Hence, a rearrangement of the outer amorphous regions is associated with the crystalline phase transition around the Curie temperature. In the literature, there are only few reports about the morphological change of P(VDF-TrFE)^30,45,55^. Supplementary Section 6 provides a comprehensive statistical analysis on the composite microstructure of P(VDF-TrFE).

**Fig. 4:**
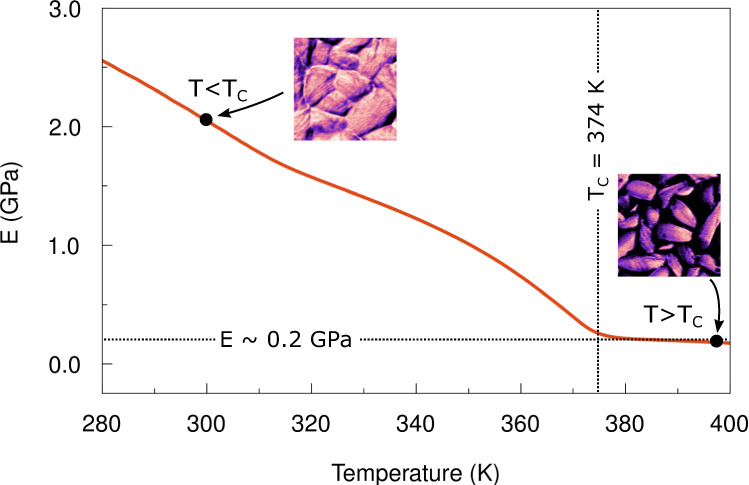
Macroscopic Young’s modulus of P(VDF-TrFE) measured by DMA. The Young’s modulus E of the bulk material P(VDF-TrFE) as a function of temperature. The insets show the Young’s modulus maps below and above the Curie temperature *T*_C_ at a corresponding macroscopic measured value, respectively.

For a deeper understanding of the composite nano- and microstructure and its impact on the measurable macroscopic material properties, we performed DMA measurements, yielding the macroscopic Young’s modulus of P(VDF-TrFE)^29^. Details of the DMA measurements are presented in Supplementary Section 7. The temperature dependency of the macroscopic Young’s modulus is shown in Fig. 4. At 300 K, we measured a Young’s modulus of 2.1 GPa, which is in good agreement with the value of 1.7 ± 0.5 GPa obtained from bimodal AFM measurements. We conclude that the crystalline domains dominate the macroscopic values of Young’s modulus measured for the ferroelectric state. The low proportion of closed amorphous islands sparsely distributed in the densely packed network of crystalline domains can be neglected.

As the temperature increases, the macroscopic Young’s modulus measured by DMA decreases linearly until it reaches a constant value of around 0.2 GPa at the Curie transition. This value is very close to the Young’s modulus measured by bimodal AFM on the amorphous regions above T_C_ (0.3 ± 0.1 GPa). Thus, we conclude that above the Curie transition the amorphous regions dominate the macroscopic mechanical response as measured by DMA. From bimodal AFM, we know that stiffer crystalline domains remain distributed inside the softer amorphous matrix. However, macroscopic mechanical measurements, e.g. DMA, do not detect them because the individual crystalline domains are completely detached from each other by an amorphous matrix.

### Micro- and macroscopic electrostrain characteristic of P(VDF-TrFE) below and above *T*_C_

**Fig. 5:**
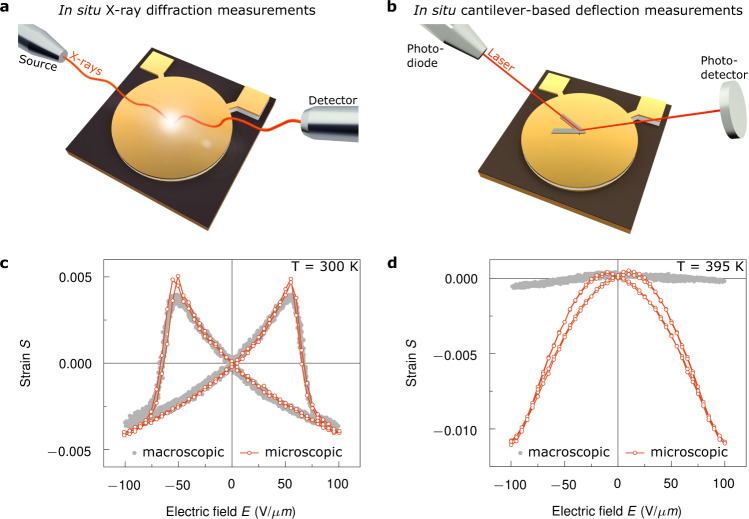
Macro- and microscopic electromechanical response of P(VDF-TrFE). **a** In situ grazing incidence XRD measurements were performed to probe the microscopic strain behaviour of the semi-crystalline P(VDF-TrFE) as a function of applied electric field. **b** To reveal the macroscopic strain behaviour of the crystalline domains during electrical stimulation, we have performed in situ cantilever-based deflection measurements. **c** Comparison of the macro- and microscopic strain behaviour *S* as a function of the applied electric field *E* for the low-temperature ferroelectric state at 300 K. **d** Comparison of the macro- and microscopic strain behaviour *S* as a function of the applied electric field *E* for the high-temperature paraelectric state at 395 K.

The embedding and separation of the crystalline domains by the amorphous polymer matrix also modifies the electromechanical properties. To demonstrate this effect, we have performed in situ grazing incidence XRD measurements, which probes the lattice spacing of the crystalline domains as a function of the applied electric field (see Fig. 5a)^17^. These measurements reveal the microscopic mechanical strain of the electroactive crystalline domains during electrical stimulation. In addition, we have compared these results with the macroscopic electromechanical response using in situ cantilever-based deflection measurements (see Fig. 5b)^35,36^. The highly sensitive optical lever readout of an AFM provides a very effective approach to probe the bulk strain behaviour of the semi-crystalline polymer P(VDF-TrFE)^56^. This method does not distinguish between the electroactive crystalline domains and the non-electroactive amorphous regions but measures the electromechanical response of the polymer as a combination of crystalline and amorphous regions. Naturally, the amorphous regions could exhibit an electrostrictive response; however, compared to crystalline materials this response is significantly smaller and can be neglected^57^. Details of the measurements performed and the extracted results are presented in Supplementary Section 8 and 9.

The macro- and microscopic strain measurements are shown in Fig. 5c for the ferroelectric state at 300 K. Both types of measurement show the same electromechanical response as a function of the applied electric field. The electrostriction coefficient $Q_{33} = - 1.9\,{\mathrm{m}}^4{\mathrm{C}}^{ - 2}$ and the piezoelectric coefficient $d_{33} = - 29.3\,{\mathrm{pm}}\,{\mathrm{V}}^{ - 1}$ obtained from the microscopic electromechanical response are in good agreement with the macroscopically determined values ($Q_{33} = - 1.7\,{\mathrm{m}}^4{\mathrm{C}}^{ - 2}$, $d_{33} = - 26.2\,{\mathrm{pm}}\,{\mathrm{V}}^{ - 1}$). At low fields, we microscopically measured a $d_{33} = - 30.5\,{\mathrm{pm}}\,{\mathrm{V}}^{ - 1}$, which agrees with the values observed above.

In the low-temperature ferroelectric state, the crystalline domains of P(VDF-TrFE) are densely packed; therefore, the macroscopic strain behaviour of the bulk material is virtually identical to that of the crystalline domains. The few amorphous islands between the densely packed crystalline domains do not contribute to the macroscopic strain behaviour.

Above the Curie transition, the macro- and microscopic strains of P(VDF-TrFE) show completely different behaviours during electrical stimulation. The bulk material exhibits almost no electromechanical response, while the crystalline domains show a strong strain as a function of the field in the high-temperature paraelectric state. At a temperature of 395 K, the micro- and macroscopic strain behaviour are shown in Fig. 5d. Although the material is in the paraelectric state, a strain of the crystalline domains of around 1% is measured. The electromechanical response is strongly electrostrictive with an electrostriction coefficient of $Q_{33} = - 10.2\,{\mathrm{m}}^4{\mathrm{C}}^{ - 2}$. However, a bistable hysteresis cycle is observed, which arises from in situ formed polar regions, as also observed as remnant polarisation in the *D*–*E* hysteresis loops above the Curie transition^32,33,58^. We also observed a dependency in the electrostrain characteristic as a function of the maximum applied field above the Curie transition (see Supplementary Section 4 for details). We propose that the deformation of the crystalline domains is compensated by the surrounding amorphous matrix, which leads to a negligible deformation for the bulk material. Above the Curie transition, the crystalline domains are embedded inside of an amorphous matrix. Consequently, the material properties of this phase dominate the macroscopic measurements. This implies that in the high-temperature paraelectric state, the Young’s modulus of the bulk material coincides with the one of the amorphous regions; in addition, the macroscopic electromechanical response corresponds to the non-electroactive amorphous phase. The crystalline domains still show a strong electroactivity; however, it is counterbalanced by the deformation of the amorphous network, which prevents its macroscopic detection. We assume that internal losses hinder the propagation of the electrostrain observed in the crystalline domains to the macroscopic level. It should be emphasised that additional effects such as clamping effects could contribute to a reduction of the macroscopic response in the high-temperature paraelectric state. However, under the same clamping conditions the crystalline domains inside of the amorphous matrix show a strong electromechanical response. Consequently, we can argue that the compensation of the macroscopic response arises from the amorphous matrix.

## Discussion

We have demonstrated that the ferroelectric polymer P(VDF-TrFE) performs a morphological phase transition driven by temperature. High-resolution bimodal AFM measurements have revealed the nano- and microstructure of the ferroelectric polymer P(VDF-TrFE) for the low-temperature ferroelectric and high-temperature paraelectric state. For the ferroelectric state, the crystalline domains are densely packed analogously to a polycrystalline material. The micro- and macroscopic electrostrain are identical. Low-field measurements of the electrostrain were in good agreement with the piezoelectric coefficients extracted from fitting the ferroelectric switching characteristic. Above the Curie transition, the crystalline domains are separated from each other, as they are embedded inside of a soft amorphous matrix. As a result, the micro- and macroscopic material properties differ significantly. The macroscopic material properties in the high-temperature paraelectric state are dominated by the amorphous phase. The crystalline domains are ‘hidden’ within the semi-crystalline microstructure of the polymer. In situ grazing incidence XRD measurements revealed a strong electroactivity of the crystalline domains on a microscopic level. However, the sub-micrometre-scale electroactive grains do not generate a macroscopic electromechanical response because the electromechanical strain of the crystalline domains is compensated by the deformation of the amorphous matrix. With our quantitative multi-scale characterisations, we have established a relationship between the polymer structure and the measured mechanical and electromechanical properties of P(VDF-TrFE), at both, the nano- and macroscales.

## Methods

### Synthesis of polymer thin films

Thin films of the copolymer P(VDF-TrFE) with a ratio between VDF and TrFE of 70 : 30 mol% were fabricated via spin-coating from polymer solutions under clean room conditions. For that purpose, a high-purity powder, purchased from the Piezotech/Arkema Group, was dissolved at a weight ratio of 8% in the solvent 2-butanone (methyl ethyl ketone (MEK)) under heating. Potential impurities of the inks were removed by filtering the solution using a filter with a pore size of 0.45 μm. To avoid air bubbles in the film, the solution was de-gassed in a vacuum desiccator. The polymer solution was coated on 4” 100 mm (100) silicon (Si) wafer, typically at 3000 r.p.m., resulting in homogeneous thin films. Residual solvent MEK was carefully evaporated at 354 K in air for 10 min. To enhance the film properties, they were annealed for 2 h in vacuum at 414 K and afterwards slowly cooled down to room temperature. The film thickness obtained was 1.0 ± 0.1 μm and was measured with a DEKTAK surface profilometer for each sample. Further information on the sample fabrication for the individual measurements is provided in Supplementary Section 2.

### Fabrication of metal-ferroelectric-metal capacitors

Micromachined capacitor-type test structures based on P(VDF-TrFE) were fabricated for the electrical and electromechanical characterisation of the polymer. The test structures are metal-ferroelectric-metal capacitors, where the ferroelectric is P(VDF-TrFE) and the electrodes/metals are made of gold (Au). We used a 4” 100 mm (100) silicon (Si) wafer coated with 150 nm LPCVD silicon dioxide (SiO_2_) as substrate. The materials used for the capacitors do not cause parasitic diffraction patterns during in situ XRD measurements. Details of the fabrication are presented in Supplementary Section 2.

### Heating system

To investigate the influence of temperature on the properties of P(VDF-TrFE), we built a miniaturised and highly precise heating system. A heating resistor (TELPOD GBR-612-12-40-1) was used as hot plate. The temperature-control unit included a PID controller and a DC power supply (HAMEG HMP2030). Separate thermocouples were used to monitor the temperature of the sample and the heating plate. The measured temperature of the sample is fed into the control unit. After 30 min of controlling the sample temperature the heat flows were in equilibrium. When this time has elapsed, the PID controller is switched off and the heating power is kept constant to avoid any current or voltage pulses. Although the control is switched off, this results in temperature fluctuations of only ±0.1 K. For the in situ XRD measurements and the DMA, we used device-specific temperature controllers. Details of the heating system are presented in Supplementary Section 5.

### Bimodal atomic force microscopy

The bimodal AFM characterisation was realised with a commercial Cypher S microscope (Asylum Research, CA, USA). The experiments were performed in dry nitrogen atmosphere. A custom-built temperature-control stage was developed to perform the AFM experiments at 300 K and 395 K (see Supplementary Section 5). The fast scan rate was fixed at 2–3 Hz. PPP-FMAuD (*f*_01_ = 56.454 kHz, *k*_1_ = 1.75 N m^−1^, *Q*_1_ = 170.1, *A*_2_ = 0.5 nm, *f*_02_ = 356.427 kHz, *k*_2_ = 69.76 N m^−1^) and PPP-FM (*A*_01_ = 71 nm, *A*_1_ = 54 nm, *f*_01_ = 79.753 kHz, *k*_1_ = 3.45 N m^−1^, *Q*_1_ = 209.3, *A*_2_ = 1.4 nm, *f*_02_ = 505.248 kHz, *k*_2_ = 176 N m^−1^) cantilevers (Nanosensors, Switzerland) have been used through all the experiments. Here we have used the bimodal AFM configuration that involves an amplitude modulation feedback for the first mode and a frequency modulation feedback for the second. The experimental output in bimodal AM-FM are used to reconstruct the nanomechanical properties of the analysed sample. Thus, a map of *I*, E_eff_ and tan *δ* can be obtained through a theoretical framework, as described in more detail in Supplementary Section 6.

### In situ cantilever-based deflection measurements

The macroscopic strain behaviour was measured in situ as a function of the applied electric field using microcantilever deflection measurements on P(VDF-TrFE) capacitors. For this purpose, the highly sensitive optical lever readout of a Bruker Dimension Edge AFM was utilised. A non-conductive silicon nitride Nanoworld PNP-TR AFM probe (*k* = 0.32 N m^−1^), which was in hard contact with the top-electrode of the capacitor, was deflected during electrical stimulation of the ferroelectric polymer. This deflection was calibrated by measuring the inverse optical lever sensitivity of the probe. To avoid significant indentation effects, the silicon wafer surface surrounding the capacitor structure was used to land the probe for the calibration factor determination. To synchronise the mechanical displacement recorded by the AFM with the triangular driving signal, a TTL trigger signal was output by the waveform generator and fed into both instruments. Details of the in situ cantilever-based deflection measurements are presented in Supplementary Section 8. In addition, the capacitor was connected in a Sawyer–Tower circuit, enabling a simultaneous recording of the dielectric displacement of the ferroelectric. The measurements were performed at 300 K and 395 K using the custom-built heating setup (see Supplementary Section 5). Details of the in situ cantilever-based deflection measurements are presented in Supplementary Section 8.

### In situ XRD measurements

The electromechanical response of the crystalline phases was recorded via quasi-static in situ grazing incidence XRD measurements. The XRD diffractometer used for our experiments was an X’Pert Pro PANalytical. The incidence angle on the sample was set to 5.0°, matching the XRD footprint to the active area of the capacitors. We applied a DC voltage to the capacitors and measured the corresponding out-of-plane scattering between angles of 2*θ* = 16.0° and 2*θ* = 24.0° in the specular plane. Repeating this procedure for different voltages, we can derive the lattice spacing of the crystalline domains as a function of the applied voltage. Each peak position was extracted via a Split Pearson VII fit to the scattered intensities. In order to improve the signal-to-noise ratio, all in situ XRD measurements were conducted in helium atmosphere. We measured the peak position with an uncertainty of about ±0.001 Å. Details of the in situ grazing incidence XRD measurements are presented in Supplementary Section 9.

## Supplementary information

Supplementary Information

## Data Availability

The data that support the findings of this study are available from the corresponding author upon reasonable request.
