# Supplementary material for: Multi-scale characterisation of a ferroelectric polymer reveals the emergence of a morphological phase transition driven by temperature
Source: Nat Commun. 2021 Jan 8;12:152. doi: 10.1038/s41467-020-20407-6 (PMC7794429; doi:10.1038/s41467-020-20407-6)
Supplement: Supplementary file 1 — Supplementary Information [file 41467_2020_20407_MOESM1_ESM.pdf]

SUPPLEMENTARY INFORMATION

Multi-scale characterisation of a ferroelectric polymer reveals the emergence of a morphological phase transition driven by temperature

By Jonas Hafner[1], Simone Benaglia[2], Filipe Richheimer[3], Marco Teuschel[1], Artner Werner[4], Franz J. Maier[1], Sebastian Wood[3], Daniel Platz[1], Michael Schneider[1], Klaudia Hradil[4], Fernando A. Castro[3], Ricardo Garcia[2] and Ulrich Schmid[1]

[1]*Institute of Sensor and Actuator Systems, TU Wien, Gusshausstrasse 27-29, 1040 Vienna, Austria*

[2]*Instituto de Cienca de Materiales de Madrid, CSIC, Sor Juana Inés de la Cruz 3, 28049 Madrid, Spain*

[3]*National Physical Laboratory, Teddington TW11 0lW, United Kingdom*

[4]*X-ray Centre, TU Wien, Getreidemarkt 9, 1060 Vienna, Austria*

Table of Contents

1. **Supplemental Introduction**……………………………………………………………...3

2. **Sample fabrication and characterisation**……………………………………………….4

3. **Microstructure of the semi-crystalline polymer P(VDF-TrFE)**………………………..9

    3.1 Morphology

    3.2 Crystal structure

4. **Ferroelectricity of P(VDF-TrFE)**……………………………………………………15

    4.1 Ferroelectric characteristic and parameter extraction

    4.2 The Curie Transition

    4.2.1 Experimental determination

    4.2.2 Crystalline phase transition

    4.3 Field-dependent electrostrain of electroactive crystals above T_c

5. **Heating system**…………………………………………………………………………..30

6. **Bimodal atomic force microscope measurements**...33

    6.1 Description of the technique and theory

    6.2 Experimental section

    6.3 Analysis and interpretation of the data

7. **Dynamic mechanical analysis**…………………………………………………………..38

8. ***In situ* cantilever-based deflection measurements**…………………………………....40

    8.1 Experimental section

    8.2 Clamping effects

9. ***In situ* X-ray diffraction measurements**…………………………….………………....48

    9.1 Experimental section

    9.2 Clamping effects

References..…...55

1. Supplemental Introduction

The supplemental information provides a more detailed description of the sample fabrication, measurements performed and the copolymer poly(vinylidene fluoride-trifluoroethylene) (P(VDF-TrFE)) investigated. We would like to provide a comprehensive insight into the measurements conducted and evaluations for the results of this work.

P(VDF-TrFE) is a ferroelectric polymer, which has been subject of ongoing research for almost fifty years. However, there are still unresolved properties of the material. Only recently, the origin of the negative piezoelectric effect was found. In particular, the semi-crystalline structure of the polymer plays a decisive role here. Supplementary Figure 1 shows the principal structure of a semi-crystalline material. Crystalline and amorphous regions interact with each other. This interaction of both mechanically and electromechanically different phases leads to unusual properties of the material.

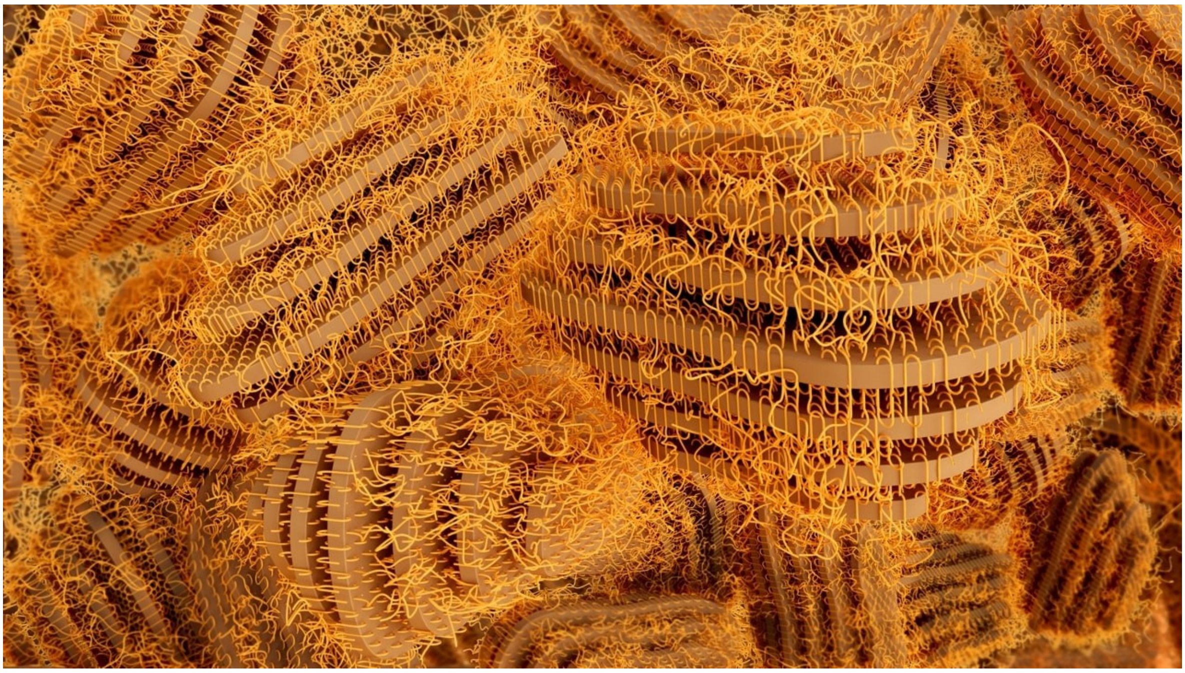

Supplementary Figure 1. Principal structure of semi-crystalline materials. Crystalline and amorphous regions form the bulk material of the ferroelectric polymer P(VDF-TrFE).

2. Sample fabrication and characterisation

Polymer thin films of P(VDF-TrFE) were fabricated under clean room conditions. For this purpose, a powder of the random copolymer P(VDF_{70}-$TrFE_{30}$) with a ratio VDF:TrFE of 70:30 mol% was purchased from the Piezotech/Arkema Group and dissolved at a weight ratio of 8% in the solvent 2-butanone (methyl ethyl ketone (MEK)). The solution was spin-coated on 4”, 100 mm (100) silicon (Si) wafers, typically at 3000 rpm. The solvent MEK was carefully evaporated at 354 K in air for 10 minutes. Subsequently, the layer was annealed for 2 hours in vacuum at 414 K and afterwards slowly cooled down to room temperature to enhance the ferroelectric properties of the polymer. The layer thickness obtained was 1.0 ± 0.1 μm and was measured with a DEKTAK surface profilometer for each sample.

Micromachined capacitor-type test structures based on P(VDF-TrFE) were fabricated for the electrical and electromechanical characterisation of the polymer. The test structures are metal-ferroelectric-metal capacitors, where the ferroelectric is P(VDF-TrFE) and the electrodes/metals are made of gold (Au). In the following, the fabrication process is described in a point-by-point manner as illustrated in Supplementary Figure 2:

(1) A 4”, 100 mm (100) Si wafer coated with 150 nm LPCVD silicon dioxide (SiO_2) was used as substrate for the capacitor-type test structures.

(2) The bottom electrodes were formed by a lift-off process. We deposited 50 nm of chromium (Cr) by electron-beam evaporation as an adhesion promoter for a 150 nm thermally evaporated Au layer.

(3) A thin film of P(VDF-TrFE) was spin-coated on the wafer and treated as previously described, resulting in a ferroelectric polymer layer with a thickness of 1.0 μm.

(4) On top of the polymer, we deposited a 100 nm thermally evaporated Au layer. A wet-chemical etching process was used to realise the top electrodes. The adhesion between Au and the polymer is sufficient, thus an adhesion layer is not necessary. We did not use a lift-off process to pattern the top electrode, since standard solvents, such as acetone, for photoresists attack the polymer.

(5) The capacitor-type test structures were completed by applying a dry etching process in O_2 plasma with the top electrode as hard mask, thus excluding any parasitic effects from the polymer film during electrical and electromechanical characterisation from the residual chip surface.

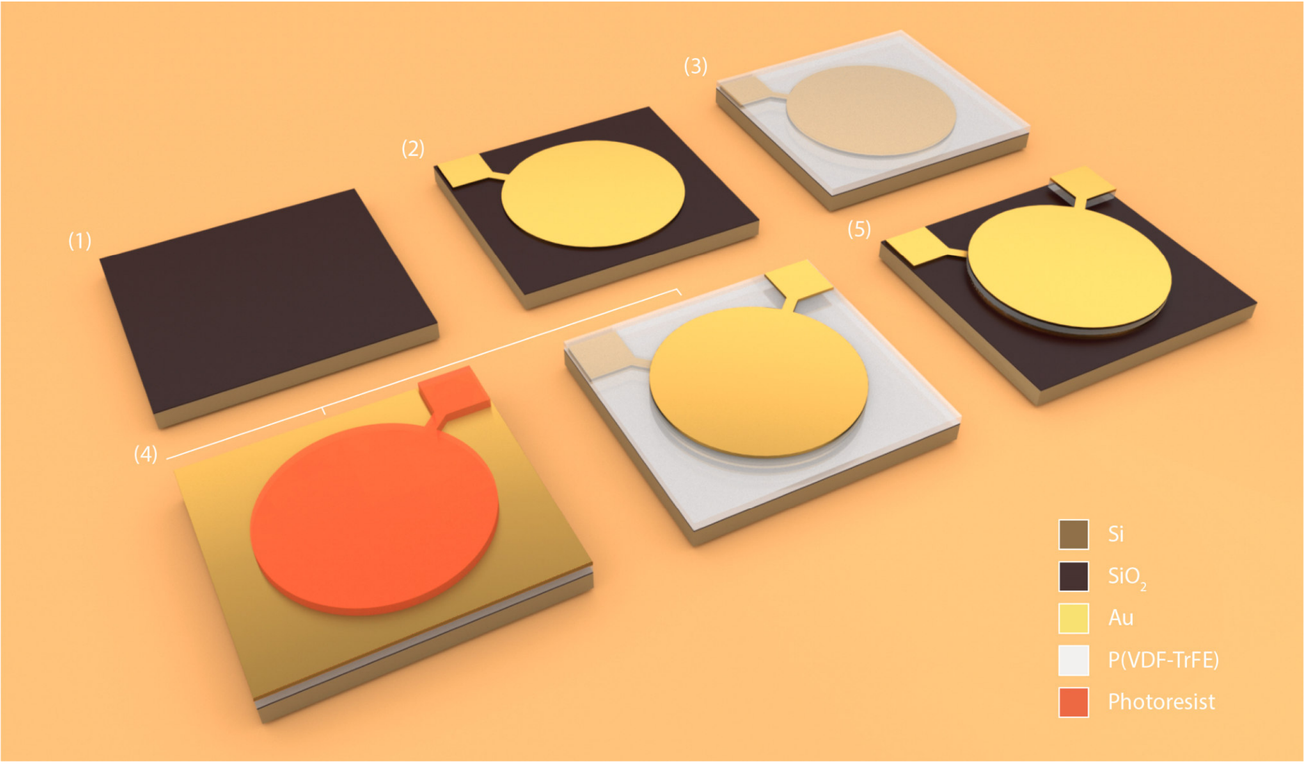

Supplementary Figure 2. Fabrication of the capacitor-type test structures. Process steps for the fabrication of the P(VDF-TrFE) based metal-ferroelectric-metal capacitors.

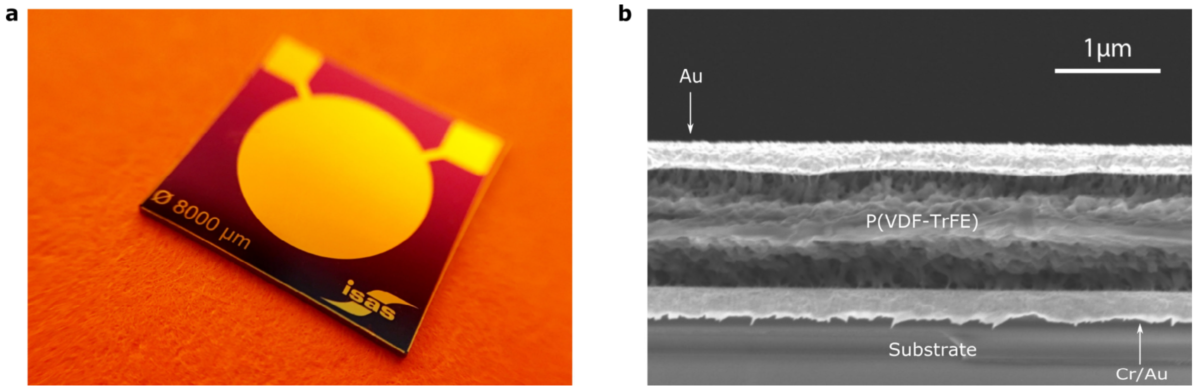

Supplementary Figure 3. Capacitor-type test structures. a, Optical photography of the resulting capacitor-type test structures with a device area of more than 0.5 cm². **b**, Cross-sectional SEM image showing the layer structure of the capacitors.

The resulting capacitor-type test structures have a device area of 0.5 cm² (see Supplementary Figure 3a). A cross section of a capacitor taken with the SEM Hitachi SU8030 is shown in Supplementary Figure 3b. The materials of the test structures are chosen such that they do not show any interference with the X-ray diffraction pattern of P(VDF-TrFE) in the measured range. This allows *in situ* grazing incidence X-ray measurements under application of an electric field to the capacitors. As the electroactive polymer is removed from the residual chip surface, parasitic effects during electric characterisation can be neglected. However, clamping effects affect electromechanical measurements, which are described in more detail in Supplementary Section 8.2.

The macroscopic polarisation of the ferroelectric polymer P(VDF-TrFE) versus electric field was measured using a Sawyer-Tower circuit, consisting of a KEYSIGHT 33600A function generator, a TREK 2100HF amplifier and a Tektronix MDO 3024 oscilloscope. We used a reference capacitor of 1 µF. Applying a 10 Hz continuous triangular wave signal to the capacitor-type test structures results in the typical switching behaviour of the macroscopic polarisation common in ferroelectrics. The dielectric constant ε_r of P(VDF-TrFE) was measured using the LCR meter Hioki IM3533-01.

In addition to the electrical and electromechanical characterisation of P(VDF-TrFE), for which the capacitors are required, measurements were also performed on polymer layers spin-coated on a silicon substrate. These include Fourier-transform infrared (FTIR) spectroscopy, specular X-ray diffraction (XRD) and atomic force microscopy (AFM) measurements. Free-standing polymer films were used for a dynamic mechanical analysis (DMA) to measure the elastic modulus of P(VDF-TrFE) bulk material. Further information on each individual measurement methodology is given in the respective chapter of the present document.

3. Microstructure of the semi-crystalline polymer P(VDF-TrFE)

3.1 Morphology

P(VDF-TrFE) is a random copolymer of the homopolymer poly(vinylidene fluoride) (PVDF). Both polymers are semi-crystalline, i.e. they consist of amorphous (disordered) and crystalline (ordered) regions. The typical morphology of a PVDF thin film (~ 300 nm) is shown in Supplementary Figure 4a. PVDF crystallises in the form of spherulites, consisting of stacks of lamellae, which grow outward from a central nucleation point. The spherulites have a diameter of several microns. The lamellae are platelet-like crystals with a thickness of about 10 nm[1-5]. The polymer chains are packed crystallographically within the lamellae, while in the gaps between lamellae they are of disordered conformation, resulting in amorphous interlamellar regions. Since a single polymer chain folds back and forth multiple times through the same lamella or across different lamellae, the crystalline and amorphous parts are strongly intertwined within a single spherulite. Between the spherulites, the polymer chains form an amorphous region. PVDF consists of about 50% amorphous parts, which are mostly located in layers between the crystalline lamellae. The crystallinity of PVDF is about 50%. For the random copolymer P(VDF-TrFE) the crystallinity increases to about 80%[6].

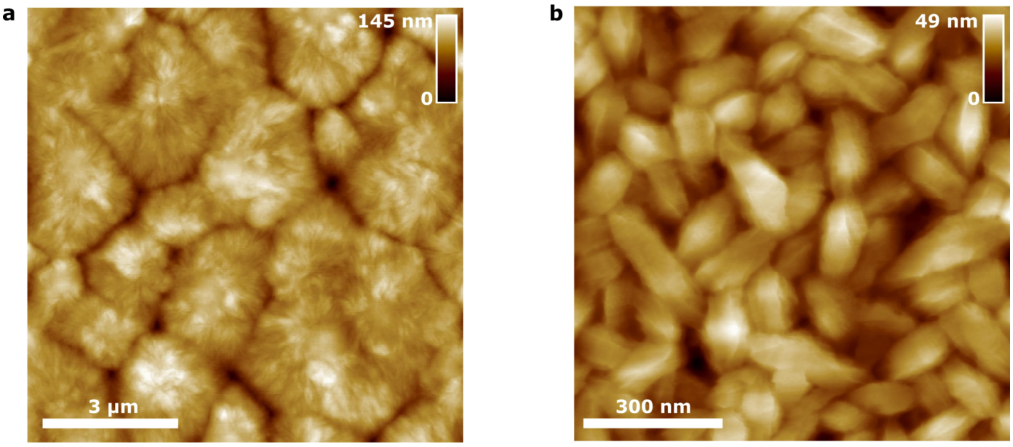

Supplementary Figure 4. AFM height images showing the morphology of PVDF and P(VDF-TrFE). a, A 300 nm thin film of PVDF crystallises into spherulitic structures. **b**, A 1 µm thin film of P(VDF-TrFE) crystallises in small rice-like domains. The AFM measurements were performed at about 300 K in air.

The morphology of a 1 μm thin film of P(VDF-TrFE) is shown in Supplementary Figure 4b. P(VDF-TrFE) crystallises in small rice-like domains. For more details about the morphology of the rice-like domains, we performed a high-resolution bimodal nanomechanical characterisation of P(VDF-TrFE) (see Supplementary Section 6). The results are shown in Supplementary Figure 5.

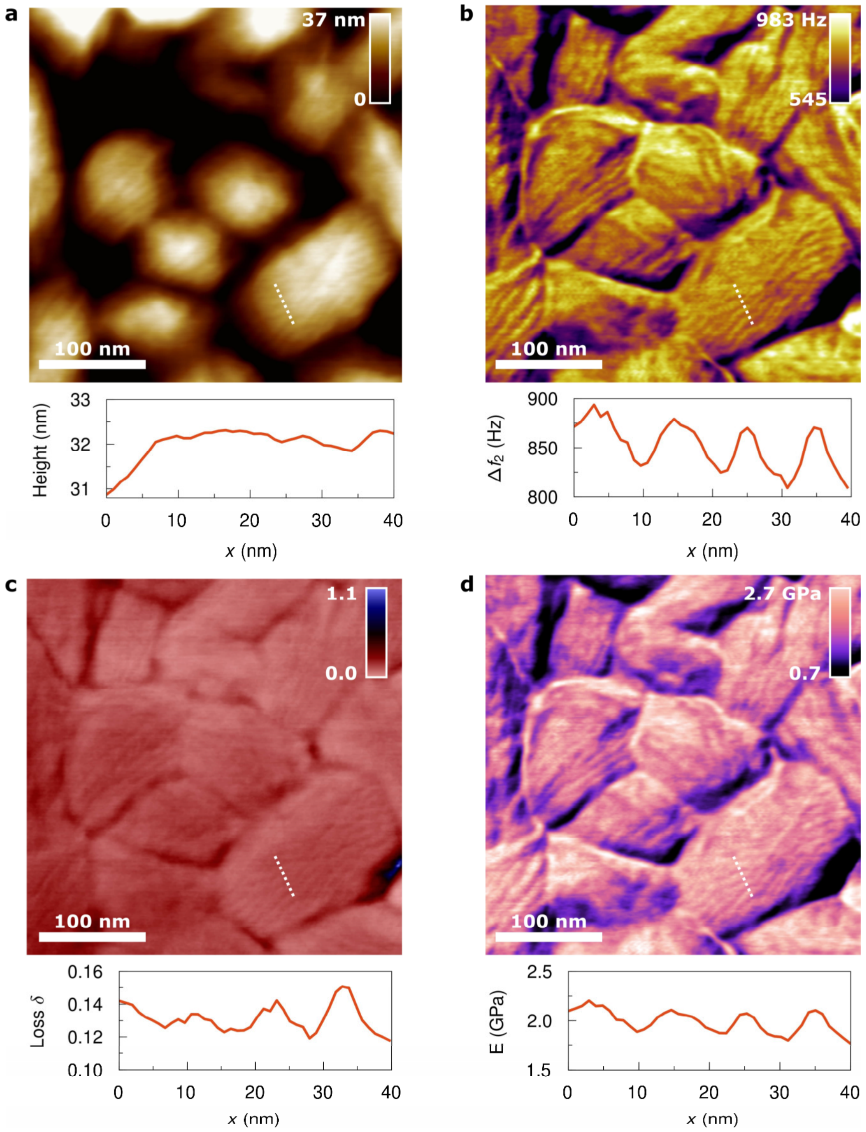

Supplementary Figure 5. High-resolution bimodal AFM images of P(VDF-TrFE). a, True topography of a 1 μm thin film of P(VDF-TrFE). **b**, Frequency shift (Δf_2) of the second mode. **c**, Loss tangent (δ) and **d**, Young's modulus (E) of P(VDF-TrFE). The profile of a cross section through the lamellae structure is shown under each image. The bimodal AFM measurements were performed at 300 K in dry nitrogen.

Similar to the spherulites of PVDF, crystalline lamellae are formed in the rice-like domains of P(VDF-TrFE). The stacks of lamellae are clearly visible in Supplementary Figure 5b. The lamellae have a thickness of about 5-8 nm and a distance of about 10 nm to each other within one single domain, which agrees with the expected results[2-5]. In between the lamellae there are amorphous regions. Crystalline and amorphous phases are strongly intermixed in P(VDF-TrFE). To distinguish the crystalline lamellae and the amorphous layers in between, we measured the loss tangent and the Young's modulus (see Supplementary Figure 5c,d). The mean value of the Young's modulus (~ 2 GPa) of the bimodal nanomechanical characterisation agrees very well with the bulk measurements of P(VDF-TrFE) (see Supplementary Section 7). For the as-cast thin films of P(VDF-TrFE), the lamellae or domains are randomly oriented. The domains are densely packed analogous to a polycrystalline material with occasional amorphous regions at grain boundaries.

3.2 Crystal structure

Inside the lamellae, the polymer chains arrange into a crystalline structure. PVDF has at least four different polymorphs known as α-, β-, γ- and δ-phase, which can be transformed into one another[6]. At ambient temperature and pressure the thermodynamically most stable structure of PVDF is the non-polar α-phase. Supplementary Figure 6a shows the chain conformation and crystal structure of the α-phase of PVDF. The non-polar nature of the α-phase is based on the fact that the unit cell consists of two *trans gauche*$^{+}$ *trans gauche*$^{-}$ ($tg^{+}tg^{-}$) chain conformations whose dipole components normal to the chain are antiparallel. However, the α-phase of PVDF can be transformed into the highly polar β-phase by biaxial stretching. To avoid such a cumbersome procedure, the copolymer P(VDF-TrFE) is often utilised. The presence of TrFE monomers destabilises the α-phase in a way that the polymer preferentially crystallises into a similar crystal structure to that of the β-phase of PVDF. The unit cell of the β-phase consists of *all trans* chain conformations whose dipole components normal to the chain are parallel, resulting in a highly polar crystal structure. The chain conformation and crystal structure of the β-phase is shown in Supplementary

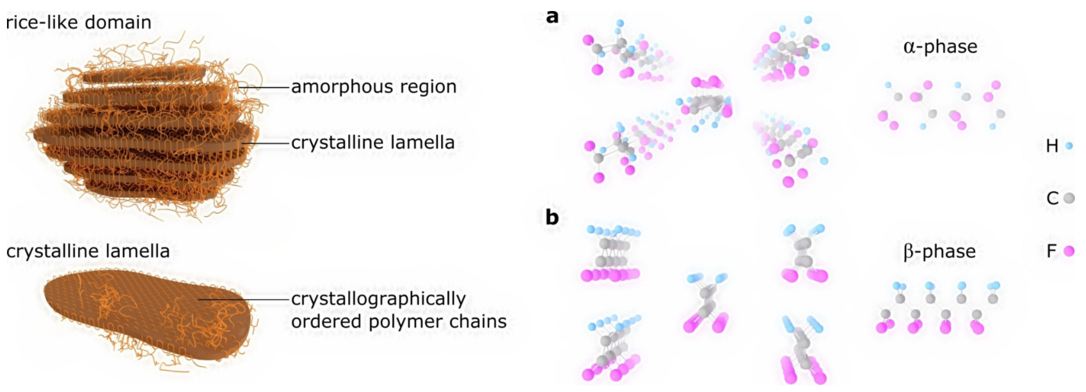

Supplementary Figure 6. Chain conformation and crystal structure of PVDF and P(VDF-TrFE). Rice-like domain and crystallographically ordered lamella of P(VDF-TrFE). **a**,**b**, α-phase and β-phase of PVDF. The copolymer P(VDF-TrFE) resembles the β-phase of PVDF under ambient conditions (see text for details).

Figure 6b. However, it should be emphasised again that the low-temperature ferroelectric state of P(VDF-TrFE) is only similar to the β-phase of PVDF, but they are not identical. The crystal structure of β-PVDF has an orthorhombic unit cell, while the low-temperature ferroelectric phase has a monoclinic unit cell. Additionally, the dimensions of the unit cells are different[2,7]. In the literature, the low-temperature ferroelectric state of P(VDF-TrFE) is often referred to as the β-phase due to the similarity of the β-phase of PVDF. However, it is important to differentiate them, instead of mixing them. Therefore, we use the nomenclature presented in ref. 7, where the ferroelectric phase (at ambient conditions) of P(VDF-TrFE) is called the low-temperature ferroelectric state. Tashiro et al. describes in ref. 7 the different phases observed for P(VDF-TrFE) for different ratios of VDF/TrFE. We highly recommend ref. 7 for an in-depth analysis of the different crystal structures and conformations observed in PVDF and P(VDF-TrFE). The δ-phase is a polar version of the α-phase, therefore also referred to as α_p-phase[8].The polar δ-phase can be obtained by poling the α-phase under a high electric field[9]. In comparison to the crystal structure of the α-phase, every second chain is rotated by 180° about its own axis, so that the chains are packed with the transverse components of their dipole moments pointing in the same direction. The δ-phase is already experimentally verified for PVDF and P(VDF-TrFE)[8-10]. We note

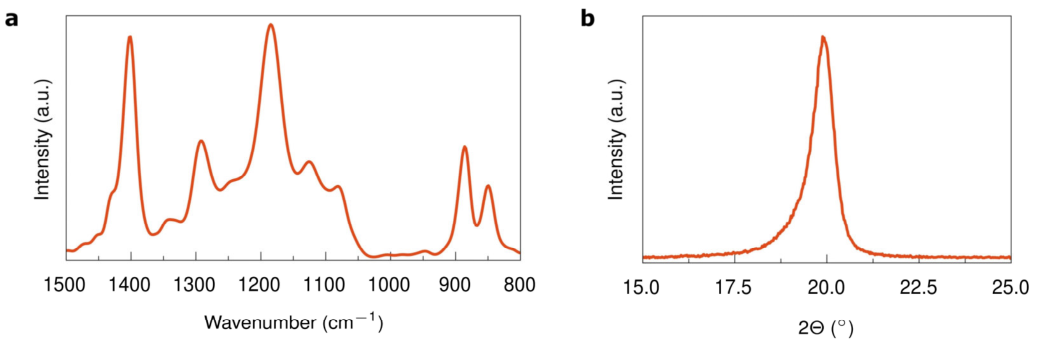

Supplementary Figure 7. Analysis of the chain conformation and crystal structure of P(VDF-TrFE). a, FTIR spectrum (absorbance) and **b**, XRD pattern of a 1 μm thin film of P(VDF-TrFE) in the low-temperature ferroelectric state. The ratio VDF:TrFE is 70:30 mol%. The measurements were performed at 300 K.

that for P(VDF-TrFE) only a phase or conformation of the polymer chains might be observed that is similar to the δ-phase of PVDF. Another chain conformation $tttg^{+}tttg^{-}$ crystallographically ordered in a polar fashion yields the γ-phase of PVDF. The γ-phase is thermodynamically unstable and therefore experimentally hardly accessible. As a final remark, we would like to emphasise again that the α-, β-, γ- and δ-phase are only specifically used for the homopolymer PVDF. The copolymer P(VDF-TrFE) may form phases that are similar to phases of PVDF, however, the chain conformation and crystal structure are generally different and the nomenclature of the phases for PVDF and P(VDF-TrFE) should be differentiated.

The fabricated P(VDF-TrFE) thin films were annealed at 414 K in vacuum for 2 hours and subsequently cooled down to room temperature (~ 300 K, see Supplementary Section 2). Under these conditions, the low-temperature ferroelectric phase is formed within the copolymer. We analysed the chain conformation and crystal structure of P(VDF-TrFE) thin films with FTIR and specular XRD measurements. The FTIR spectrum in Supplementary Figure 7a shows two strong absorption bands at 1288 cm^{-1} and 850 cm^{-1}, intrinsic to the CF_2 vibration and CH_2 rocking modes of the *all trans* conformation that is characteristic for the low-temperature ferroelectric phase or the β-phase conformation[11,12]. The formation of the crystal structure of the low-temperature ferroelectric phase was confirmed by specular XRD measurements, as

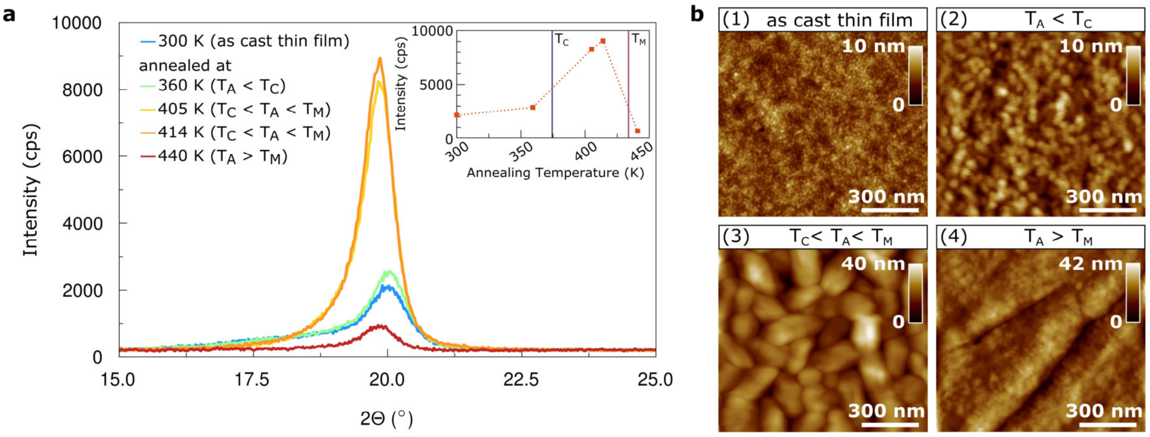

Supplementary Figure 8. Impact of the annealing temperature on the crystallinity of P(VDF-TrFE). a, XRD patterns of 1 μm thin film of P(VDF-TrFE) annealed at different temperatures. The inset shows the peak intensity as a function of the annealing temperature. **b,** Morphology of P(VDF-TrFE) annealed at different temperatures: (1) as cast thin film without any thermal treatment, (2) annealed at T_A = 360 K, (3) annealed at T_A = 414 K, (4) annealed at T_A = 440 K. The ratio VDF:TrFE is 70:30 mol%. The Curie temperature (T_C) is around 374 K and the melting temperature (T_M) is around 432 K. The measurements were performed at 300 K in air.

shown in Supplementary Figure 7b, in which the characteristic diffraction peak at a 2θ value of 19.7° is evident for P(VDF_{70}-$TrFE_{30}$). This diffraction peak is attributed to the (110) and (200) orientation plane of the pseudohexagonal crystal structure of the low-temperature ferroelectric phase[12]. In the literature, a modification of the low-temperature phase was found in P(VDF-TrFE), called the cooled phase[2,6,7,13]. However, after thermal annealing between the Curie temperature (T_C) and the melting temperature (T_M), where the so-called high-temperature phase is formed, only the low-temperature phase remains in the polymer after cooling. Additionally, we note that the ratio VDF:TrFE is 70:30 mol%; here, the low-temperature phase is in general formed at ambient conditions. We also note that the cooled phase is typically observed for low VDF content in P(VDF-TrFE). In ref. 7, the cooled phase is described in more detail and we recommend the reader to have a look at this reference. Furthermore, the crystallinity and consequently the ferroelectric properties of PVDF and P(VDF-TrFE) strongly depend on the annealing temperature. Supplementary Figure 8a shows specular XRD measurements of P(VDF-TrFE)

annealed at different temperatures. Above T_C the thermal energy allows the polymer chains to rearrange resulting in a more crystalline structure after cooling. If the polymer is annealed above T_M almost no *all trans* chain conformation (or β-phase) is present at room temperature. Hence, an annealing temperature of 414 K being between T_C and T_M is optimal for a high degree of crystallinity (see inset of Supplementary Figure 8a). These results are validated by AFM measurements of the polymer. The impact of the annealing temperature on the morphology is shown in Supplementary Figure 8b. For the as-cast thin film (the solvent evaporation step was omitted) and the film annealed at $T_A < T_C$ only very small domains are observable. Rice-like domains are formed for $T_C < T_A < T_M$, which collapse when the film is annealed above T_M. P(VDF_{70}-$TrFE_{30}$) has a Curie temperature of about 374 K and a melting temperature of about 432 K[6].

In general, the properties of PVDF and P(VDF-TrFE) are sensitive to the fabrication process. For instance, electron-irradiation strongly affects the electrical and electromechanical properties resulting in a relaxor ferroelectric behaviour[14]. Nanoconfined polymer films show a similar behaviour (known as the nanoconfinement effect)[15,16]. Also the amount of TrFE monomers in P(VDF-TrFE) has a strong impact on the Curie and melting temperatures. Overall, the properties of both polymer and copolymer can be affected by a variety of fabrication processes. However, PVDF and P(VDF-TrFE) are still semi-crystalline polymers. Functional thin films studied in this work, such as those fabricated according to Supplementary Section 2, are always of the same structural nature. And it is actually their semi-crystalline morphology that makes them different from conventional ferroelectric materials like barium titanate ($BaTiO_3$), which have a continuous three-dimensional solid crystal structure.

4. Ferroelectricity of P(VDF-TrFE)

4.1 Ferroelectric characteristic and parameter extraction

Polar materials in which the spontaneously generated electric polarisation can be reversed by inverting an external electric field are called ferroelectrics. Consequently, ferroelectrics exhibit two stable polarised states in the absence of an electric field and the application of an electric field can induce switching between these states. This polarisation bistability can be measured experimentally by the canonical polarisation hysteresis curve. Supplementary Figure 9a shows the electric displacement *D-E* hysteresis loops of the ferroelectric P(VDF-TrFE) measured with the capacitors fabricated according to Supplementary Section 2. The hysteresis loops are divided into four coloured segments, with the electric field sequence indicated by the arrows and numbers. Starting from a negative polarised film, the polarisation switches to a positive orientation with increasing electric field at the coercive field (E_c) and finally starts to saturate at higher voltages (see segment 1 in Supplementary 9a). A subsequent decrease of the voltage results in a slight reduction of the saturated polarisation up to the remnant polarisation (P_r) at zero field (see segment 2 in Supplementary Figure 9a). Segments 3 and 4 show the inverse process of the segments 1 and 2. To extract parameters like E_c or P_r, we use an empirical model developed by Miller et al. to fit the ferroelectric *D-E* hysteresis loops[17].

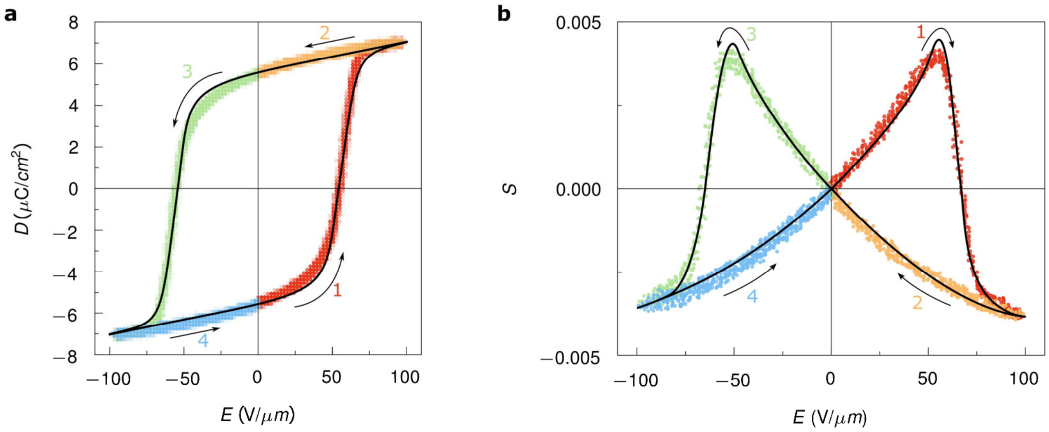

Supplementary Figure 9. Polarisation and strain characteristics of P(VDF_{70}-$TrFE_{30}$). a, Ferroelectric displacement (*D-E* hysteresis) and **b**, macroscopic strain (*S-E* butterfly curve) as a function of the applied field. The solid lines are fits according to the proposed models. The measurements were performed at 300 K.

Supplementary Table 1. Fit parameters for the ferroelectric characteristics of P(VDF-TrFE).

Material	E_c (V/µm)	P_r (µC/cm²)	Q_{33} (m⁴/C²)	d_{33} (pm/V)
P(VDF$_{70}$-TrFE$_{30}$)	**55**	**5.8**	**-1.7**	**-26.2**

Here, we measured D between the bottom and top electrode of the capacitors in z-direction. An electric field E with a frequency of 10 Hz was applied to the electrodes. The ferroelectric displacement $D = D(E)$ is the sum of the induced polarisation $P_i = P_i(E)$ and the spontaneously generated polarisation $P_s = P_s(E)$:

$$D = \varepsilon_0 E + P = \varepsilon_0 E + P_i + P_s = \varepsilon_0 \varepsilon_r E + P_s. \qquad \text{(S1)}$$

For a fit of P_s, we have to distinguish the two parts composing the hysteresis loop. For instance, the hysteresis branch P_s^+ with the field ramping from negative to positive is given by:

$$P_s^+(E, E_{max}) = P_{max} \tanh\left(\frac{E-E_c}{\delta}\right) + \frac{P_{max}}{2}\left(\tanh\left(\frac{E_{max}+E_c}{\delta}\right) - \tanh\left(\frac{E_{max}-E_c}{\delta}\right)\right). \qquad \text{(S2)}$$

In equation (S2), E_{max} is the maximum applied field, P_{max} is the maximum polarisation and δ is given by:

$$\delta = 2E_c\left(\ln\left(\frac{1+\frac{P_r}{P_{max}}}{1-\frac{P_r}{P_{max}}}\right)\right)^{-1}. \qquad \text{(S3)}$$

Similarly, the other branch P_s^- with the field ramping from positive to negative is given by the relation:

$$P_s^-(E, E_{max}) = -P_s^+(-E, E_{max}). \qquad \text{(S4)}$$

The fitted curves of the hysteresis loops are shown in Supplementary Figure 9a and the resulting fit parameters are presented in Supplementary Table 1. We note that this method has been widely used to model the ferroelectric displacement D as a function of the field E[17-20].

As mentioned above, ferroelectrics are a subset of polar materials, which generate an electric field under an applied mechanical stress and vice versa. Given this relationship, ferroelectrics exhibit piezoelectricity. The macroscopic electrostrain S in P(VDF-TrFE) is shown in Supplementary Figure 9b. The strain is calculated from the *in situ* cantilever-based deflection measurements (see Supplementary Section 8) as the relative change in thickness of the polymer film between the two electrodes of the capacitors:

$$S = \frac{\Delta l}{l_0} \tag{S5}$$

In equation (S5), Δl is the change in the thickness of the polymer film from its zero field value l_0 in z-direction, i.e. $S = S_3$. In a phenomenological theory, it was shown that the origin of piezoelectricity in PVDF and its copolymer P(VDF-TrFE) is electrostriction[21]. Hence, the strain S can be expressed by a Taylor series expansion of the displacement D_3 with the first non-vanishing term

$$S = S_3 = Q_{33}D_3^2 = Q_{33}(\varepsilon_0\varepsilon_r E + P_s)^2 = Q_{33}\varepsilon_0^2\varepsilon_r^2E^2 + 2Q_{33}\varepsilon_0\varepsilon_r P_s E + Q_{33}P_s^2, \tag{S6}$$

where Q_{33} is the longitudinal electrostriction coefficient written with two indices following Voigt`s notation. The electrostriction is a quadratic effect and a universal property of all crystal symmetries due to the anharmonicity of the chemical bonds. The first term, $Q_{33}\varepsilon_0^2\varepsilon_r^2E^2$, on the right-hand side of equation (S6) corresponds to the pure electrostrictive effect. The second term, $2Q_{33}\varepsilon_0\varepsilon_r P_s E$, is referred to as the linear longitudinal piezoelectric effect with the piezoelectric coefficient

$$d_{33} = \frac{\partial S}{\partial E}\bigg|_{E=0} = 2Q_{33}\varepsilon_0\varepsilon_r P_r. \tag{S7}$$

Hence, the piezoelectric effect in ferroelectric crystals arises from the electrostrictive effect biased by its spontaneous polarisation. We note that d_{33} is the effective piezoelectric constant (see Supplementary Section 8.2). In general, most ferroelectrics exhibit a positive piezoelectric effect, i.e. they expand by

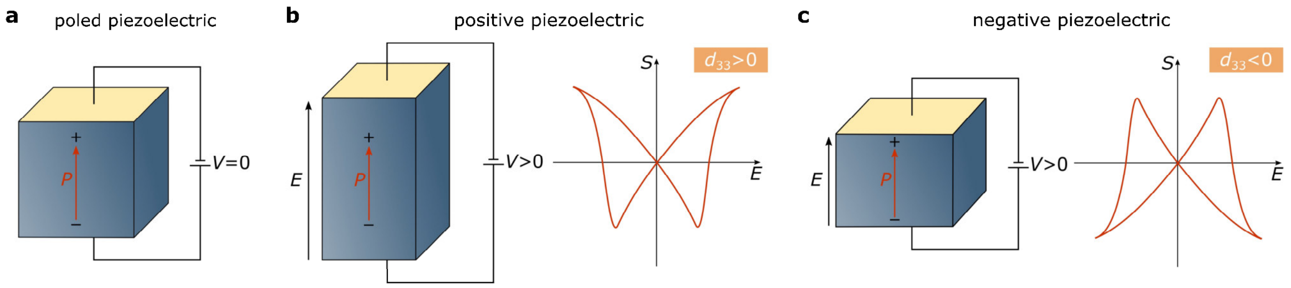

Supplementary Figure 10. Positive and negative piezoelectric effect. **a**, Initial state of a poled piezoelectric. **b**, Positive piezoelectric: Applying an electric field along the direction of the polarisation results in an expansion of the material (e.g. lead zirconate titanate (PZT)). **c**, Negative piezoelectric: Applying an electric field along the direction of the polarisation results in a contraction of the material (e.g. PVDF).

applying an electric field along the existing polarisation direction (see Supplementary Figure 10b). In contrast, negative piezoelectrics contract under this applied field condition (see Supplementary Figure 10c). PVDF and its copolymers are negative piezoelectrics due to their one-dimensional molecular chains, bonded together by weak van der Waals forces[20]. In Supplementary Table 1, we show the fit parameter Q_{33} of the *S*-*E* characteristic, often called a butterfly curve, (see Supplementary Figure 9b), and the resulting piezoelectric coefficient d_{33}. For the calculation of d_{33} according to equation (S7), we used $\varepsilon_r = \varepsilon_r(E = 0) = 15$, which was measured using a commercial LCR meter (Hioki IM3533-01). In our measurements, the extracted values of the electrostriction and piezoelectric coefficient are in good agreement with those reported in literature[19-21]. The last term, $Q_{33}P_s^2$, on the right-hand side of equation (S6) represents the spontaneous strain. If there is no external electric field applied to the ferroelectric, the strain is zero. However, in analogy to the remnant polarisation, ferroelectrics have a remnant strain corresponding to $Q_{33}P_r^2$. Thus, the strain has been set to zero at zero electric field.

In the following, we would like to go into details about the fitting procedure according to equation (S6). Furukawa and Seo showed that in ferroelectric polymers piezoelectricity arises from electrostriction and found that the ferroelectric *S*-*E* characteristic can be described as the square of the *D*-*E* hysteresis loop[21].

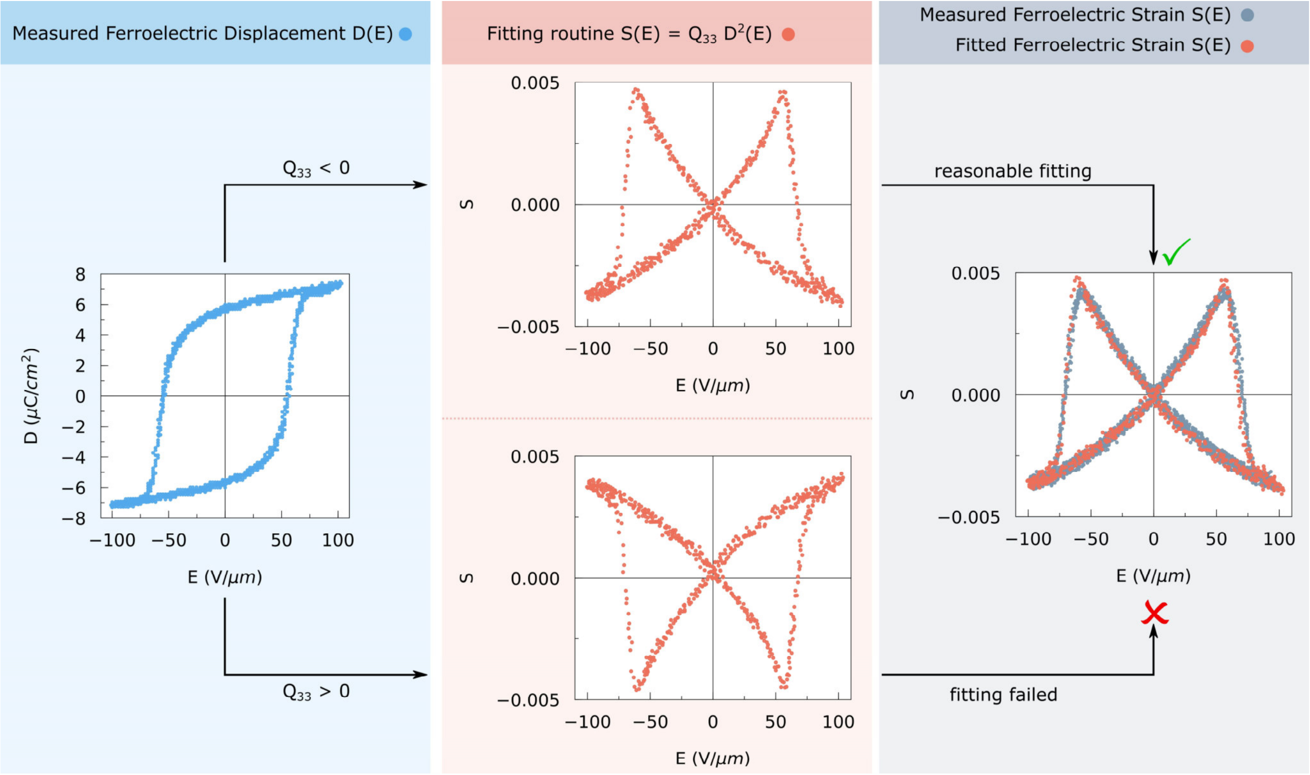

Supplementary Figure 11. Fitting procedure of the ferroelectric *S-E* characteristic. Starting from the entire *D-E* hysteresis loop measured with a Sawyer-Tower circuit, we fit the measured *S-E* characteristic according to $S(E) = Q_{33}D^2(E)$. The fitting parameter is the longitudinal electrostriction coefficient Q_{33}. For P(VDF-TrFE), a negative $Q_{33} = -1.7\ \mathrm{m}^4/\mathrm{C}^2$ is extracted with a R-Squared of 0.922. Additionally, we show that a positive Q_{33} runs into a fitting failure, meaning that for $Q_{33} < 0$ a negative R-Squared of -0.09 is obtained, e.g. the fit is worse than a horizontal line. The fitting routine was realised in Python using SciPy Curve Fit.

The factor of proportionality between *S* and *D*^{2} represents the longitudinal electrostriction coefficient Q_{33}. Consequently, Q_{33} is the parameter to find, which fits best the relation between the measured *S-E* and *D-E* data according to $S(E) = Q_{33}D^2(E)$. The fitting procedure is illustrated in Supplementary Figure 11. According to equation (S6), the measured *D-E* and *S-E* data are required for the fitting routine. For that purpose, we used the entire *D-E* hysteresis loop and the entire *S-E* characteristic of P(VDF-TrFE). We note that due to symmetry in the *S-E* curve it would be sufficient to consider only a half cycle of the field *E*, meaning a half cycle either from minimum to maximum field (segment 4 and 1) or from maximum

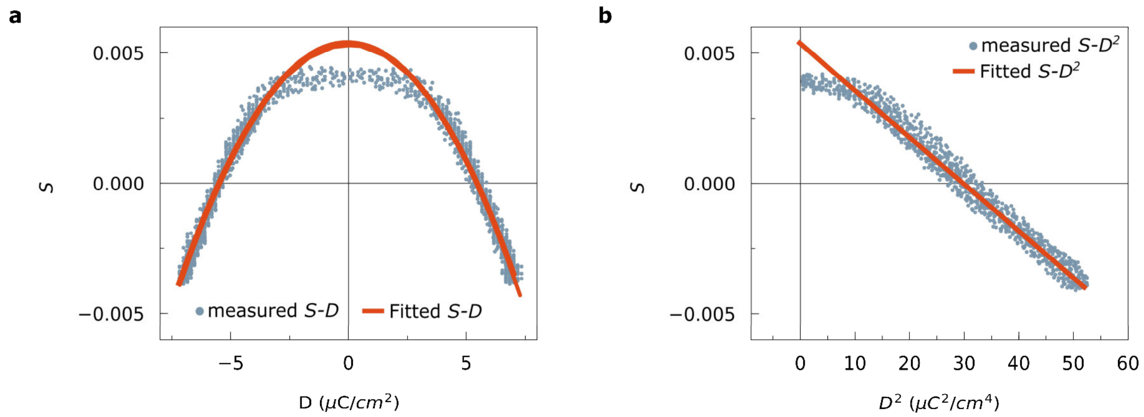

Supplementary Figure 12. Strain characteristic as a function of *D* and *D*^{2}. The measured electromechanical strain *S* is plotted as a function of (**a**) the ferroelectric displacement *D* and (**b**) the squared ferroelectric displacement *D*^{2}. For comparison, the fitted results are presented, respectively.

to minimum field (segment 2 and 3). However, only using one segment of the *D-E* and *S-E* data is incorrect and we recommend to use the entire set of data. The fitting routine was then realised in Python using SciPy Curve Fit. The result is presented in Supplementary Figure 11. Additionally, we show the resulted fit as a function of *D* and *D*^{2} in comparison to the measured *S-D* and *S-D*^{2} data in Supplementary Figure 12, which are the same as described by Furukawa and Seo[21]. For P(VDF-TrFE) a negative $Q_{33} = -1.7\ \mathrm{m}^4/\mathrm{C}^2$ is extracted with a R-Squared of 0.922. Consequently, P(VDF-TrFE) exhibits a negative piezoelectric effect (compare with Supplementary Figure 10c). To corroborate that only a negative Q_{33} can describe the measured *S-E* characteristic, we limited Q_{33} to a positive range ($Q_{33} > 0$) and tried to fit the measured *S-E* data. However, the fitting failed. In Supplementary Figure 11, we can see that in that case $Q_{33} \rightarrow 0$ and a negative R-Squared of -0.09 is obtained, meaning that a fit with a positive Q_{33} is worse than a horizontal line. There is no way to describe the entire *S-E* curve with a positive Q_{33}.

To extract the negative piezoelectric constant d_{33} of P(VDF-TrFE) from the extracted Q_{33}, we rewrite the ferroelectric displacement *D(E)* in equation (S6) as the sum of the induced polarisation $P_i(E)$ and the

spontaneous polarisation $P_s(E)$, which are both functions of the field E and not a constant[19,20]. Doing so, we yield the right-hand side of equation (S6). We would like to note, that in equation (S6) also ε_r is a function of the field. However, for the calculation of d_{33}, as described in equation (S7), we evaluate all field-dependent parameters at zero field $E = 0$, meaning that the permittivity $\varepsilon_r(E = 0) = \varepsilon_r = 15$ and the spontaneous polarisation $P_s(E = 0) = P_r = 5.8\ \mu\mathrm{C/cm^2}$ are constant values. Consequently, a negative piezoelectric constant of $d_{33} = -26.2\ \mathrm{pm/V}$ is extracted.

In addition to the extraction of d_{33} by fitting the nonlinear ferroelectric switching dynamics of P(VDF-TrFE), we directly measured d_{33} at low electric fields ($E_{\mathrm{max}} < E_c = 55\ \mathrm{V/\mu m}$) for comparison. In that case a linear relation between the electromechanical strain S and the electric field E is expected. The factor of proportionality between S and E is then the piezoelectric constant d_{33}. We note that the sign of the slope between S and E depends on the sign of the remnant polarisation. For instance, a positive polarised film with $P_r^+ = +5.8\ \mu\mathrm{C/cm^2}$ exhibits a negative slope, while a negative polarised film with $P_r^- = -5.8\ \mu\mathrm{C/cm^2}$ exhibits a positive slope. In that way, the unique switching characteristic between two polarisation states of ferroelectric materials can be tested and observed in electrostrain measurements at low fields.

To measure the electrostrain at low fields, we used the *in situ* cantilever-based deflection measurement setup, which is described in detail in Supplementary Section 8. Since the expected deflection for a thin film at low fields is less than 1 nm, we measured the noise level of the setup beforehand. A noise level of approximately ± 0.2 nm at a bandwidth of 5 kHz for each datapoint ensures trustful results from the setup even at low fields.

Before performing the measurements at low fields, we polarised P(VDF-TrFE) in one direction. We applied a DC field to the film of 100 V/μm for 10 min to positive polarise the ferroelectric (see Supplementary Figure 13a). Afterwards, we suddenly turned off the DC field, ensuring that the film

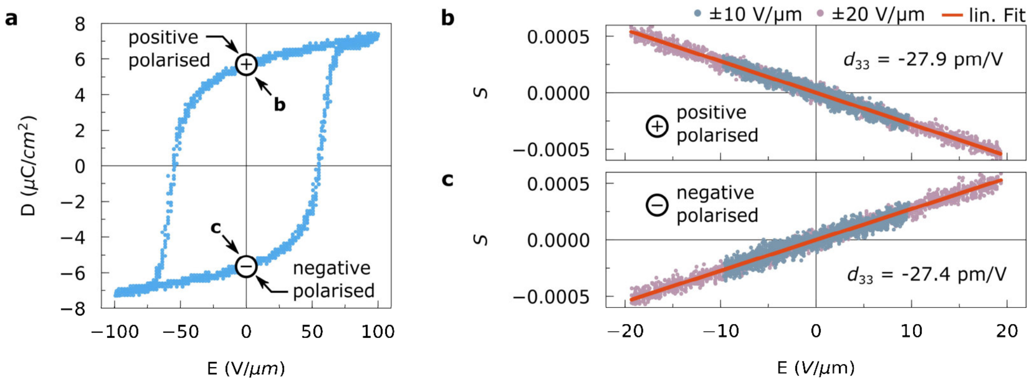

Supplementary Figure 13. Low-field strain measurements. a, The slope of the strain at low fields depends on the direction of the polarisation. The ferroelectric P(VDF-TrFE) exhibit two polarisation states: positive and negative polarised. **b**,**c**, Strain versus field for P(VDF-TrFE) at fields below the coercive field (±10 V/µm, ±20 V/µm). The piezoelectric constant d_{33} was extracted via linear fitting.

remained polarised in that direction, and started with the electrostrain measurements at low fields below the coercive field to avoid any switching between the two polarisation states. To that end, a triangular signal at a frequency of 10 Hz was applied to the film with an amplitude of ±10 V/µm or ±20 V/µm. In Supplementary Figure 13b, the strain S of a positive polarised film is plotted as a function of the field E. As expected, a linear relation is measured. The piezoelectric coefficient can be extracted via linear fitting of the S-E characteristic. We extract d_{33} = -27.9 pm/V. To demonstrate the switching behaviour of the film, we applied a DC field of -100 V/µm for 10 min to the positive polarised film in order to switch the polarisation in the negative direction. Afterwards, we repeated low-field measurements. The strain versus field for a negative polarised film is shown in Supplementary Figure 13c. Indeed, we switched the polarisation direction, since the slope of the electrostrain switched the sign. We extract d_{33} = -27.4 pm/V. We note that the slope of the S-E characteristic is always directed in the opposite direction of the polarisation state, verifying the negative piezoelectric nature of P(VDF-TrFE). The low-field measurements show that the extraction of d_{33} via nonlinear fitting of the ferroelectric switching at high fields provide trustful results. Both methodologies yield the same piezoelectric constant.

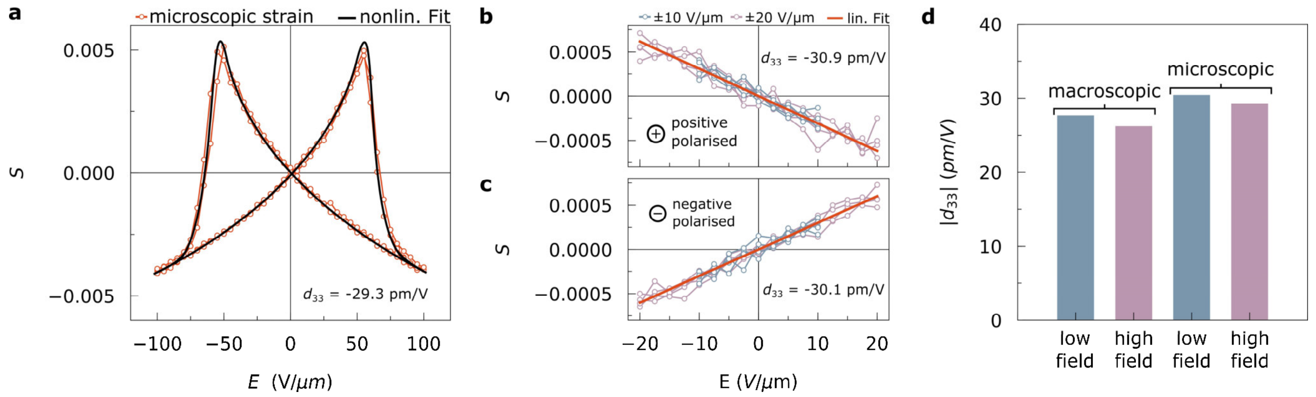

Supplementary Figure 14. Microscopic electrostrain characteristic. a, Microscopic butterfly curve of P(VDF-TrFE) at room temperature and the resulting fit. **b**,**c**, Microscopic strain versus field for P(VDF-TrFE) at fields below the coercive field (±10 V/µm, ±20 V/µm). The piezoelectric constant d_{33} was extracted via linear fitting. **d**, Comparison of all extracted piezoelectric constants $|d_{33}|$ of the microscopic and macroscopic electrostrain at low and high fields. The piezoelectric constants presented were measured in the low-temperature phase.

We note that the strain data presented here are related to macroscopic measurements, showing the strain behaviour of the bulk material of P(VDF-TrFE) under the influence of an external field. The microscopic strain data obtained from the *in situ* X-ray diffraction measurements at high fields are presented in detail in Supplementary Section 9. The microscopic butterfly curves are fitted in the same way as presented in this section. In Supplementary Figure 14a, we present the microscopic *S-E* curve of P(VDF-TrFE) measured at high fields, showing the characteristic butterfly shape. A nonlinear fit of the microscopic *S-E* characteristic yields an electrostriction coefficient of $Q_{33} = -1.9\ \mathrm{m^4/C^2}$ and a piezoelectric constant of $d_{33} = -29.3\ \mathrm{pm/V}$. To complete the several electrostrain measurements presented, we measured the microscopic electrostrain at low fields analogously to the macroscopic electrostrain measurements at low fields. The results are shown in Supplementary Figure 14b,c. A piezoelectric constant of about $d_{33} = -30.5\ \mathrm{pm/V}$ is extracted. A comparison of all piezoelectric constants extracted from the various measurement methodologies is given in Supplementary Figure 14d. The independent measurements provide similar piezoelectric constants, corroborating the measured d_{33}.

4.2 The Curie transition

Ferroelectricity is not observable at all temperatures. At higher temperatures, ferroelectrics may change to a centrosymmetric lattice or develop a disordered structure with randomly oriented dipoles. In this range, conventional ferroelectrics lose almost all of the macroscopic remnant polarisation, leading to a paraelectric behaviour in the material. The temperature at which the transition from the ferro- to paraelectric state occurs, is called the Curie temperature T_C.

4.2.1 Experimental determination

The Curie transition can be determined experimentally via the *D-E* hysteresis at different temperatures. For this purpose, we measured via the Sawyer-Tower circuit (see Supplementary Section 2) the *D-E* hysteresis loops at various temperatures using a custom-built heating system (see Supplementary Section 5). Supplementary Figure 15a shows the temperature-dependent remnant polarisation of P(VDF-TrFE). As the temperature increases, the remnant polarisation initially remains almost constant until it decreases significantly at a temperature of around 370 K. A change from a single to a double hysteresis loop is displayed (see Supplementary Figure 15b), resulting from the coexistence of the

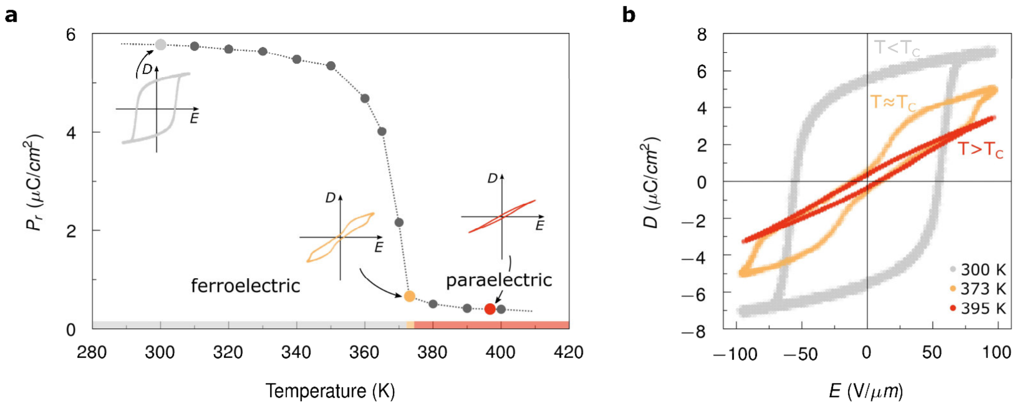

Supplementary Figure 15. Thermal evolution of the ferroelectric characteristics of P(VDF-TrFE) a, Remnant polarisation P_r as a function of temperature. The dashed line is a guide for the eye. **b**, *D-E* hysteresis loops at temperatures below, around and above the Curie temperature T_C, showing the ferroelectric and paraelectric characteristics of P(VDF-TrFE). The ratio VDF:TrFE is 70:30 mol%.

para- and ferroelectric phase. The double hysteresis loop is a feature of a first-order phase transition, previously reported for P(VDF-TrFE)[22,23]. Above a temperature of 374 K, the double hysteresis vanishes. Thus, the Curie temperature is T_C = 374 K, which is in good agreement with reported values in literature for a ratio VDF:TrFE of 70:30 mol%[6,22-26].

We note that the Curie transition strongly depends on the ratio between VDF and TrFE in the copolymer P(VDF-TrFE)[6,23]. The Curie temperature increases with the VDF mole fraction. For instance, the homopolymer PVDF does not show a distinct Curie transition, since melting takes place before phase transition.

It is striking, that P(VDF-TrFE) exhibits a narrow single hysteresis loop in the high-temperature paraelectric phase (see Supplementary Figure 15b). At a temperature of 395 K, which is more than 20 K higher than the Curie temperature, a remnant polarisation $P_r \approx 0.4\ \mu C\ cm^{-2}$ is still observable. In Ref. 11 and 12, this phenomenon was justified by the induction of both nucleation and growth of ferroelectric domains under a sufficiently high electric field. The remnant polarisation results from *in situ* formed polar regions under high-field poling. We see this ferroelectric-like response also in the microscopic strain-field behaviour of the high-temperature paraelectric phase of P(VDF-TrFE) (see Supplementary Section 4.3). Additionally, we note that narrow single hysteresis loops are common for relaxor ferroelectrics[4,14-16,27,28].

4.2.2 Crystalline phase transition

For a better understanding of the Curie transition, we analysed the crystal lattice of P(VDF-TrFE) as a function of temperature via specular XRD measurements. The heating system and temperature control unit used are presented in Supplementary Section 5. Supplementary Figure 16a shows the temperature dependence of the lattice spacing *d* in a temperature range from 300 K to 400 K. Below the Curie transition, the crystal is best represented as long *all trans* sequences, which is consistent with the FTIR spectrum measured at 300 K (see Supplementary Figure 16b). With increasing temperature, the XRD

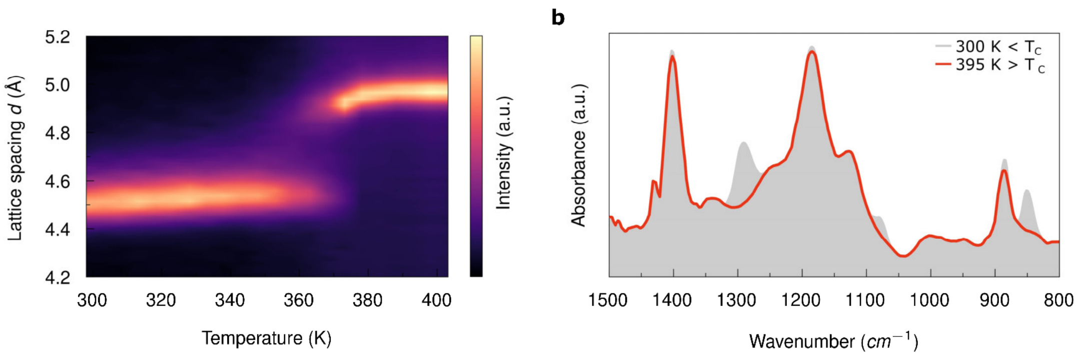

Supplementary Figure 16. Crystalline phase transition of P(VDF-TrFE). a, Specular XRD measurements showing the lattice spacing *d* as a function of temperature. **b**, FTIR measurements below and above the Curie transition. The ratio VDF:TrFE is 70:30 mol%.

pattern changes from the low-temperature ferroelectric state into the high-temperature paraelectric phase[2,7,28-44]. The coexistence of the ferro- and paraelectric phase below the Curie transition, as can be seen in Supplementary Figure 16a, induces the double *D-E* hysteresis loop of the electrical measurements in Supplementary Section 4.2.1. At a temperature of $T = T_C = 374$ K, the characteristic XRD peak of the low-temperature ferroelectric state vanishes, indicating the Curie transition of the material. The FTIR spectra in Supplementary Figure 16b provides the same results; the *all trans* sequences are almost vanished above T_C and a mixture of *gauche* bonds is present. In detail, the crystalline change from the ferro- to paraelectric phase is caused by intramolecular changes through the introduction of $gauche^+$ and $gauche^-$ conformations into the *all trans* structure of the low-temperature phase of P(VDF-TrFE). The paraelectric phase of P(VDF-TrFE) is a statistical combination of tg^+, tg^-, $tttg^+$ and $tttg^-$ rotational isomers and the unit cell shows a high sliding motion along the c-axis[2,7,35-37]. The irregularly distributed short *trans* sequences above the Curie transition are remains of the TrFE segments of P(VDF-TrFE). In the literature, the high-temperature paraelectric phase of P(VDF-TrFE) is often referred to as the α-phase, meaning that the paraelectric phase of P(VDF-TrFE) resembles the α-phase of PVDF. However, this is incorrect. The polymer chains in the high-temperature paraelectric phase of P(VDF-TrFE) do not have the well-defined tg^+tg^- conformation as in the α-phase, but a random conformation with a high sliding motion along the

c-axis of the unit cell. In addition, the conformation is not fixed at all and the twisted conformation rotates and changes very fast[7]. Therefore, the high-temperature paraelectric state is not even similar to the α-phase of PVDF. At this point, we highly recommend again to read ref. 7 that describes in detail the Curie transition characteristic of P(VDF-TrFE). Nonetheless, we note that results reported in literature for the Curie transition characteristics of P(VDF-TrFE) are in good agreement with those presented in this section[2,7,28-44].

Additionally, we note that the Curie transition naturally depends on a continuously applied electric field[40,41]. For instance, applying a strong bias field during heating a polymer film of P(VDF-TrFE) would lead to an increased Curie temperature. However, the field-dependent measurements in this work were performed at elaborated temperatures either below or above the Curie transition. We did not apply a continuous field to the polymer film during heating, which would influence the Curie transition. The XRD patterns of the *in situ* XRD measurements in Supplementary Section 9 corroborate that the polymer film passed the Curie transition, since only the characteristic XRD pattern of the paraelectric phase (also during field stimulation after heating the sample) was present.

4.3 Field-dependent electrostrain of electroactive crystals above T_c

Above the Curie transition of P(VDF-TrFE), we observed a field-dependent electromechanical response of the crystalline domains. Field-induced phase transitions in the high-temperature paraelectric state of P(VDF-TrFE) are not new and were already observed in literature[15,16,38-43]. For instance, it was shown that the high-temperature paraelectric phase of P(VDF-TrFE) can be transformed into a ferroelectric phase by applying a sufficiently high electric field. A field-induced growth of polar regions above the Curie transition is often attributed to an intramolecular change through an increase of the short *trans* sequences along the chain axis with increasing field. However, it should be noted that at high temperatures and under high-field poling, ferroelectric domains form *in situ*, meaning that they are short-lived and largely

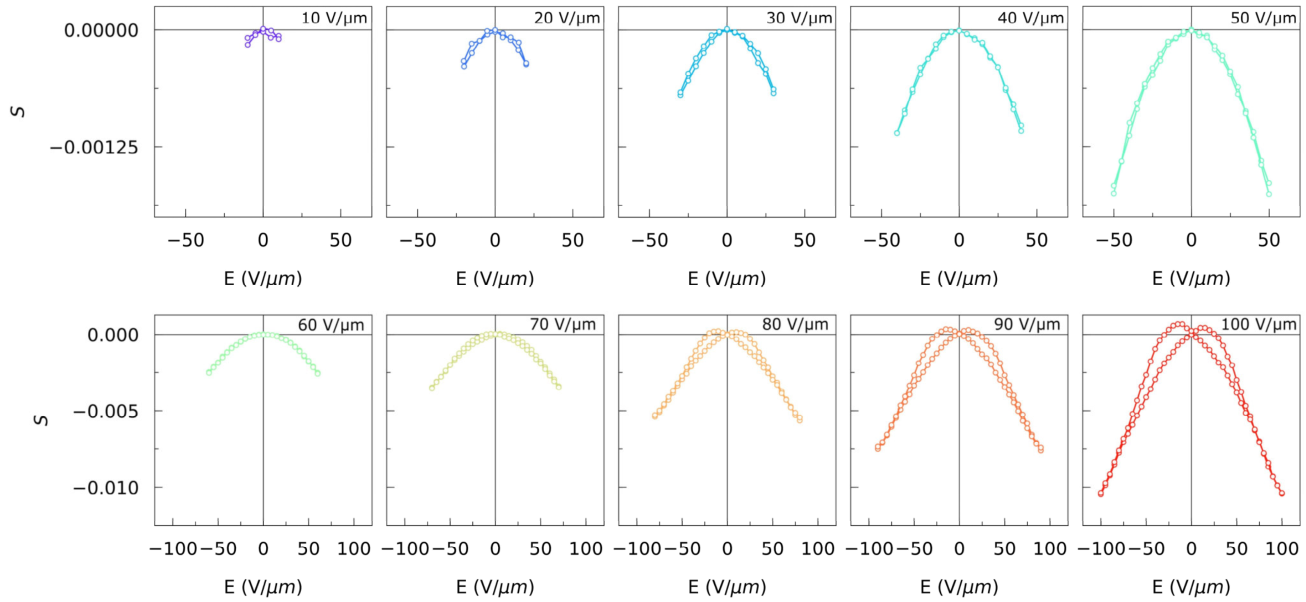

Supplementary Figure 17. Field-dependent electrostrain characteristics above T_C. Microscopic electrostrain of the high-temperature paraelectric phase as a function of the applied field *E*. A transition from a pure electrostrictive to a bistable hysteretic response in the *S-E* characteristic can be observed, indicating a field-induced phase transition in the intramolecular structure of P(VDF-TrFE).

disappear after removing the poling electric field. To that end, we investigated in detail the impact of an electric field on the high-temperature paraelectric phase of P(VDF-TrFE). To observe any field-dependent change in the electromechanical response of the paraelectric phase, we measured the microscopic *S-E* characteristics of the paraelectric XRD peak as a function of the maximum applied electric field. It should be emphasised that we did not apply any voltage to the sample during heating, which would influence the Curie transition. The field-dependent XRD measurements were performed at an elaborated temperature above the Curie transition. We studied the impact of an electric field on the behaviour of the paraelectric phase, which is corroborated by the fact that we measured the periodical shift of the characteristic XRD peak of the paraelectric phase. For detailed information about the measurement methodology, we refer to Supplementary Section 9.

Supplementary Figure 17 shows a series of the microscopic electrostrain *S* as a function of the field *E* for an increasing value of the maximum applied field. We did not apply any field to the paraelectric phase before conducting the field-dependent measurements, ensuring that the pure paraelectric phase was present. Hence, it was possible to observe the impact of the electric field on the paraelectric phase by increasing successive the maximum applied field. We see that the crystalline domains in the paraelectric phase of P(VDF-TrFE) exhibit a pure electrostrictive response, which is characteristic for the high-temperature paraelectric state. However, starting from an electric field of around 80 V/µm a bistable hysteretic response in the electrostrain starts to occur. For 100 V/µm, we can clearly see the hysteresis in the *S*-*E* characteristic. Obviously, a growth of polar regions is induced by the electric field in the high-temperature paraelectric phase of P(VDF-TrFE); this effect was also observed in the *D*-*E* hysteresis loops measurements above the Curie transition. It should be noted that these polar regions induced by an electric field in the high-temperature paraelectric phase of P(VDF-TrFE) may arise from local *all trans,* $tttg^{+}$ *or* $tttg^{-}$ conformations. However, it is expected that these polar regions are short-lived and largely disappear after removing of the poling field. Nonetheless, a clear field-dependency in the electrostrain characteristic of the high-temperature paraelectric phase can be observed, that is in good agreement with the *D*-*E* hysteresis loops measurements.

5. Heating system

In order to investigate the influence of temperature on topography, morphology, electrical, mechanical and electromechanical properties, samples must be temperature loaded under defined conditions. Therefore, the heating unit is a key component, enabling a most precise temperature control of both the sample and the hotplate, respectively. The core tasks are temperature control and monitoring of the sample and hotplate temperature. To that end, a custom-built heating system was designed to allow high controllability and stability of the sample temperature. In addition, a hotplate with a low thermal mass was selected to have a low influence on the temperature during the measurements performed. To minimise heat transfer to the stage the heating element is placed on a ceramic plate[45,46].

Device specific temperature controllers were used for XRD (Anton Paar TCU100) and DMA (TA Instruments DMA Q800) measurements. Supplementary Figure 18 shows the heating system for the bimodal AFM measurements, respectively, with the temperature control unit, heating stage and temperature monitoring unit. The temperature control unit includes a PID controller realised in Python and a DC power supply (HAMEG HMP2030). A heating resistor (TELPOD GBR-612-12-40-1) was used as the heating stage. In addition, we monitored the evolution of temperature during the entire time of the measurements. From the control loop in Supplementary Figure 18, the basic controller principle is presented. The desired measurement temperature T_{set} is specified as the reference variable. The control deviation ΔT results from the difference between the desired temperature T_{set} and the current sample temperature T_{sample}. The actuating value, which represents the electrical power, is determined from the calculated control deviation. A change in the electrical power leads to current and voltage pulses. Various disturbances, such as temperature flows towards the sample holder, thermal expansions, ambient temperature, air circulation and temperature dependent components, affect the controlling system. The actual temperature of the sample and the heating plate are determined by individual thermocouples

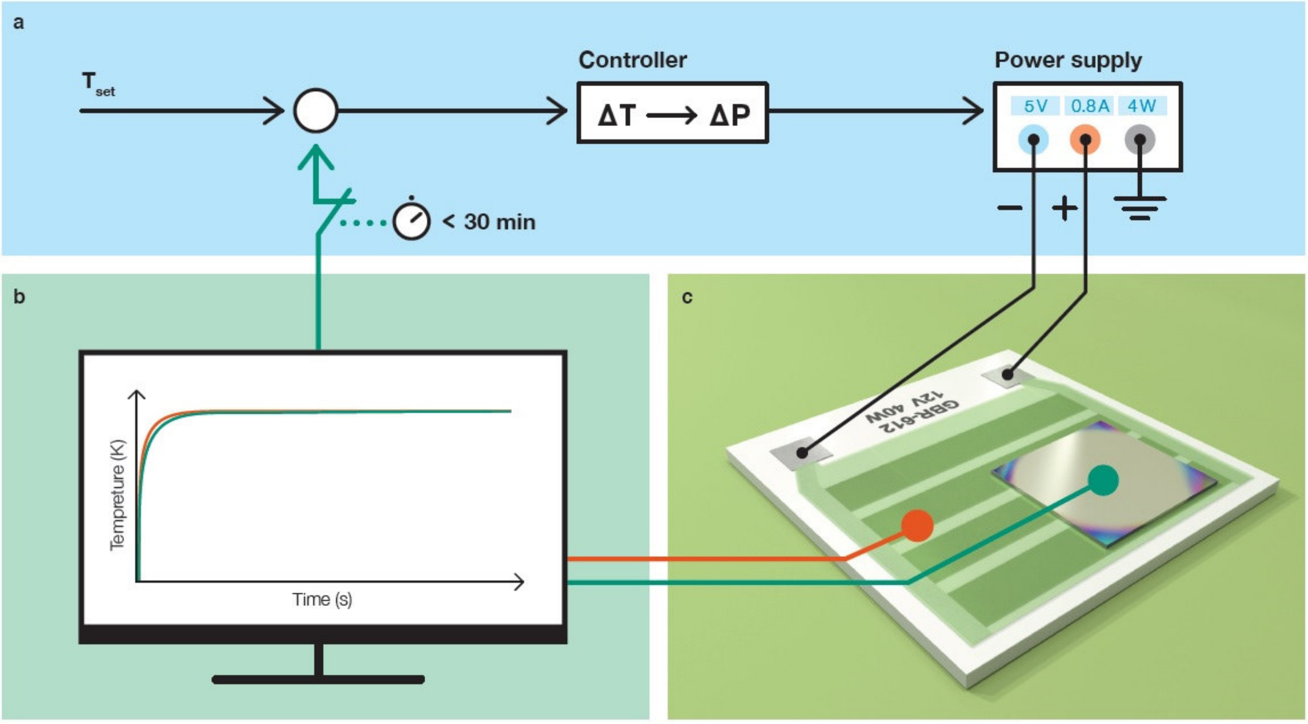

Supplementary Figure 18. Diagram for the used heating system. a, Temperature control unit with the PID controller and the DC power supply for the hotplate. **b**, Temperature monitoring unit showing the temperature characteristics of the hotplate (red) and the sample (green). **c**, The hotplate used with a P(VDF-TrFE) sample on it.

thermally separated. The temperature of the sample is then fed into to the control unit. After 30 minutes of controlling the sample temperature the heat flows are in equilibrium. When this time has elapsed, the PID control is switched off and the heating power is kept constant, so that there are no more current or voltage pulses. The regulator has been designed to avoid overshoot in the temperature. When approaching the setpoint temperature, the heating power is reduced. In order to avoid even the smallest current or voltage pulses, these two values are kept constant after 30 minutes, resulting in lower measurement noise. Although the control is switched off, this only leads to temperature fluctuations of ± 0.1 K, as shown in Supplementary Figure 19. Additionally, it shows that the difference between sample and hotplate temperature is only 0.25 K. We note that we did also bimodal AM-FM AFM measurements up to temperatures of around 450 K, and even at such high temperatures stable conditions for nanomechanical

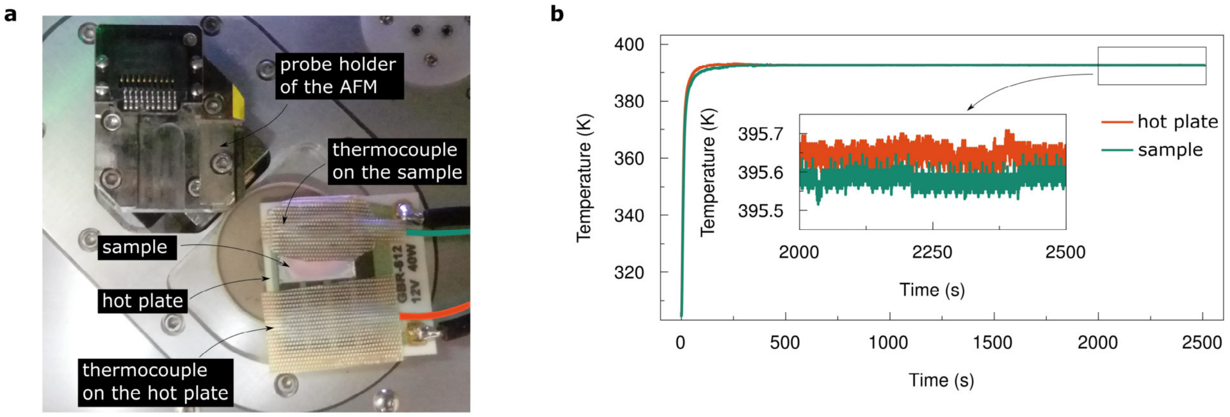

Supplementary Figure 19. Temperature control unit for the bimodal AFM measurements. a, Photo of the used bimodal AFM setup. **b**, Typical temperature characteristics of the hot plate (red) and the sample (green).

mapping measurements of the sample surface were accessible. However, only our system ensured such stable conditions for nanomechanical mapping. To that end, we highly recommend the reader to perform AM-FM AFM measurements as presented here. We want to emphasise that we are willing to give more details on the heating control upon on request (please contact the corresponding author).

6. Bimodal atomic force microscope measurements

6.1 Description of the technique and theory

To understand the effect of temperature on the morphological and nanomechanical properties of the polymer P(VDF-TrFE), we have performed high resolution characterisation measurements with the bimodal AFM.

Bimodal AFM[47] is an advanced dynamic AFM method where maps of the topography and mechanical properties of a surface are recorded with atomic or nanometre-scale spatial resolutions depending on the sample stiffness. In this case we have used the bimodal AM-FM configuration[48,50]. In this configuration the first two flexural resonances of the microcantilever are excited simultaneously. Thus, the first mode and the second mode are controlled, respectively, by amplitude (AM) and frequency modulation (FM) feedbacks. The total deflection of the cantilever can be approximated by[49]

$$z(t) = z_0 + z_1(t) + z_2(t) \approx A_1 \cos(2\pi f_1 t - \phi_1) + A_2 \cos(2\pi f_2 t - \phi_2), \quad \text{(S8)}$$

where z_0, z_1 and z_2 are the static deflection and the deflection of both modes while A_1, A_2, f_1, f_2, ϕ_1 and ϕ_2 are the amplitudes, the driving frequencies and phase shifts of the two modes. During imaging, the feedbacks operate as follows. The amplitude of the first mode is set to the set-point amplitude (A_1), which is smaller than the free-oscillation amplitude A_{01}; A_2 is set constant and ϕ_2 is kept at $\pi/2$ by means of shifting f_2 at the resonance (Δf_2). The quantitative theory of bimodal AFM requires that the value of A_2 should be much smaller than the one of A_1. The tip-surface force is modelled following the Hertz contact mechanics:

$$F_{ts} = (4/3) E_{\text{eff}} \sqrt{R_t I^3}, \quad \text{(S9)}$$

where I is the indentation of the sample, R_t the radius of the tip and E_{eff} is the effective Young's modulus of the sample. Using the approximation $E_{tip} >> E_s$, where E_s is the Young's modulus of the sample, the

effective Young's modulus of the sample is

$$E_{\text{eff}} \approx \frac{E_s}{(1-\nu_s^2)}, \tag{S10}$$

where ν_s is the Poission coefficient of the sample (in our case $\nu_s = 0.34$). In bimodal AM-FM, I and E_{eff} are linked to the observables by

$$I = \left(\frac{A_{01}k_1}{2Q_2k_2}\right)\frac{\cos\phi_1}{(\Delta f_2/f_{02})}, \tag{S11}$$

$$E_{\text{eff}} = \left(\sqrt{\frac{8A_1}{R_t}}\right)\left(\frac{\Delta f_2}{f_{02}}\right)^2 \frac{2Q_1k_2^2}{\text{k}_1\text{A}_{01}\cos\phi_1}, \tag{S12}$$

where k_1 and k_2 are the force constants of the first and the second mode and Q_1 is the Q-factor of the first mode. The loss tangent is determined by

$$\tan\delta = \frac{G''}{G'} = \frac{sin\phi_1 - A_{01}/A_1}{cos\phi_1}, \tag{S13}$$

which is a dimensionless parameter that measures the ratio between the dissipated energy (*G''*) and the stored energy (*G'*)[51].

6.2 Experimental section

The bimodal AFM measurements are performed using a commercial microscope, Cypher S AFM (Asylum Research, CA, USA). The experiments are performed at ambient condition ($T_0 \approx 300$ K) in a dry nitrogen atmosphere. The custom-built heating system is implemented inside the microscope. The temperature is controlled as detailed in Supplementary Section 5. We then perform bimodal nanomechanical characterisation at two different temperatures, in such a way to distinguish the transition below and above the Curie temperature T_C. The line scan rate was fixed at 2-3 Hz. PPP-FMAuD and PPP-FM cantilevers (Nanosensors) have been used throughout all the experiments. The calibrated parameters are reported in the caption of each image. The first mode of the cantilever is calibrated through

the Asylum research GetReal^{TM} tool, allowing calibration of the cantilever stiffness at the beginning of the experiment. Since bimodal AFM is usually based on the use of the first and the second mode of the cantilever, the calibration of the second mode is a critical step. The second mode of the cantilevers is calibrated by assuming the stiffness-frequency power law relationship, which is given by

$$k_2 = k_1 \left(\frac{f_2}{f_1}\right)^{\zeta 2}, \tag{S14}$$

where ζ_2 is an experimental calibration parameter[51]. In bimodal AM-FM, the apparent topography, A_1, ϕ_1 and Δf_2 are recorded simultaneously. Thus, the last three observables are used to derive the nanomechanical parameters I, E_{eff} and tan δ. Eventually, the deformation I is used to reconstruct the true topography of the sample, simply adding I to the apparent topography. The radius of the tip is estimated by using a test sample made of PS-b-PMMA (poly(styrene-block-methyl methacrylate))[48]. The radius of the tip lies in the range of 6 nm to 12 nm, and remains constant during a single experiment. The experiments were repeated with different cantilevers and different samples to confirm the reproducibility of the data. In addition, we have performed sequential experiments with the same tip to verify the measurement stability during bimodal AM-FM data acquisition.

6.3 Analysis and interpretation of the data

Supplementary Figure 20 shows Young's modulus maps of the polymer P(VDF-TrFE) at two temperatures (300 K and 395 K). The average value of the Young's modulus decreases at the higher temperature, which lies above the Curie transition. Cross-sections are shown in the bottom panels. The mean value of elastic modulus in the amorphous regions remains approximately constant, while the Young's modulus and the topography on the grains have changed. The images indicate that the amorphous regions are rearranged by increasing the temperature. The lamella structures observed at room temperature, tend to disappear above the Curie temperature T_C (Supplementary Figure 20c,d), although

some of them are still present at 395 K. This indicates that crystalline domains are still present in the high-temperature paraelectric phase.

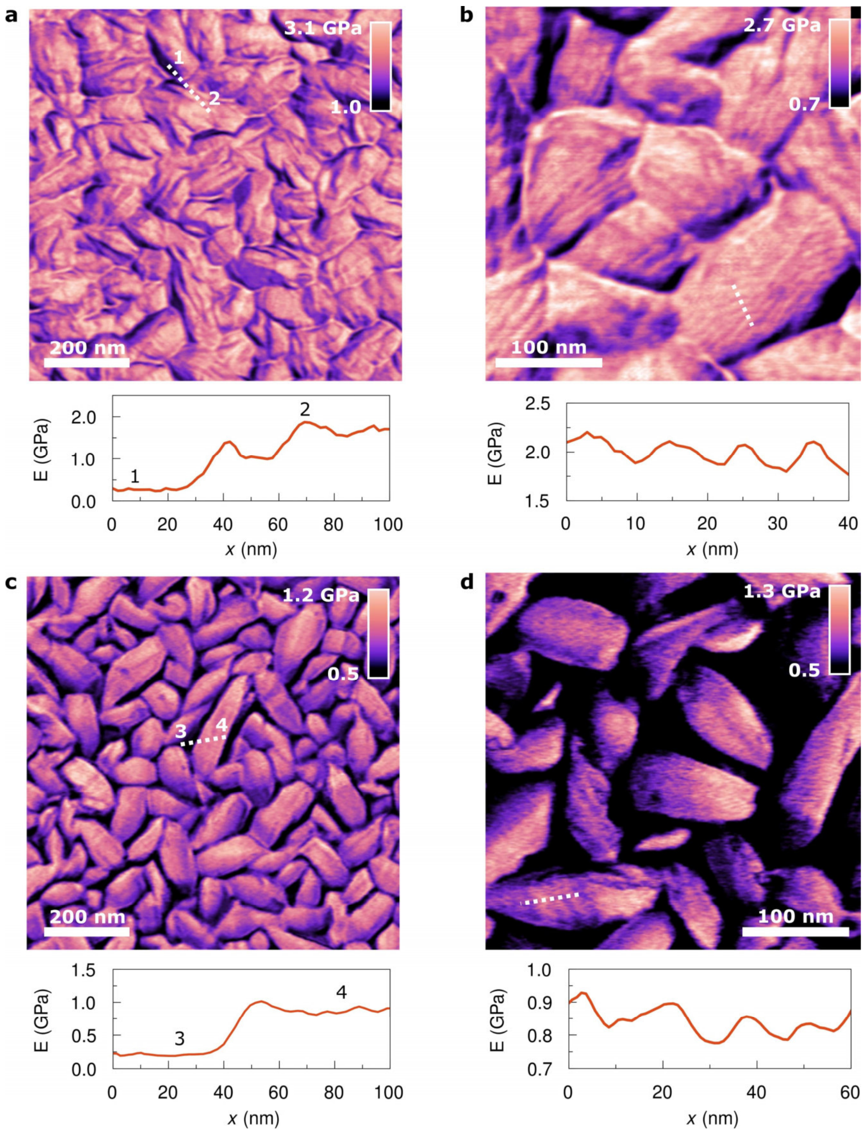

Supplementary Figure 20. Young's modulus maps of P(VDF-TrFE) below and above T_C. a,b, Young's modulus maps of a P(VDF-TrFE) thin film recorded at 300 K. **c,d,** Young's modulus maps of a P(VDF-TrFE) thin film recorded at 395 K. The bottom panels show the cross-sections along the lines marked in the images. Lamellar structures are observed below and above the Curie temperature T_C. Bimodal AM-FM data: A_{01} = 110 nm, A_1 = 74 nm, f_{01} = 56.454 kHz, k_1 = 1.75 N m^{-1}, Q_1 = 170.1, A_2 = 0.5 nm, f_{02} = 356427 kHz, k_2 = 69.76 N m^{-1} (panels a, c and d). A_{01} = 71 nm, A_1 = 54 nm, f_{01} = 79.753 kHz, k_1 = 3.45 N m^{-1}, Q_1 = 209.3, A_2 = 1.4 nm, f_{02} = 505.248 kHz, k_2 = 176 N m^{-1} (panel b).

To quantify the above observations, we have performed a statistical analysis to characterise the reorganisation of the polymer as a function of temperature (see Supplementary Figure 21). First, we have determined the Young's modulus threshold value at a location of a crystalline phase on the surface. This is done by determining the Young's modulus of the crystalline regions at a temperature of 395 K, where the amorphous regions have a higher related fraction (see Supplementary Figure 20). The regions of the polymer with a Young's modulus value lower than the threshold for a crystalline region are assigned to the amorphous regions. The threshold is obtained by subtracting the standard deviation of the mean value of the crystalline part (Gaussian fitting of the data (Supplementary Figure 21c)). Then, a mask with the defined threshold is applied on each image (Supplementary Figure 21a,b). This step provides the number of pixels assigned as amorphous, which are normalised with respect the total number of pixels of each image. We have performed the analysis on 8 images taken on different samples with different cantilevers. At 300 K the percentage of pixels assigned to amorphous regions is 2.5% ± 0.9%, while at 395 K this is significantly higher (37.7% ± 3.8%).

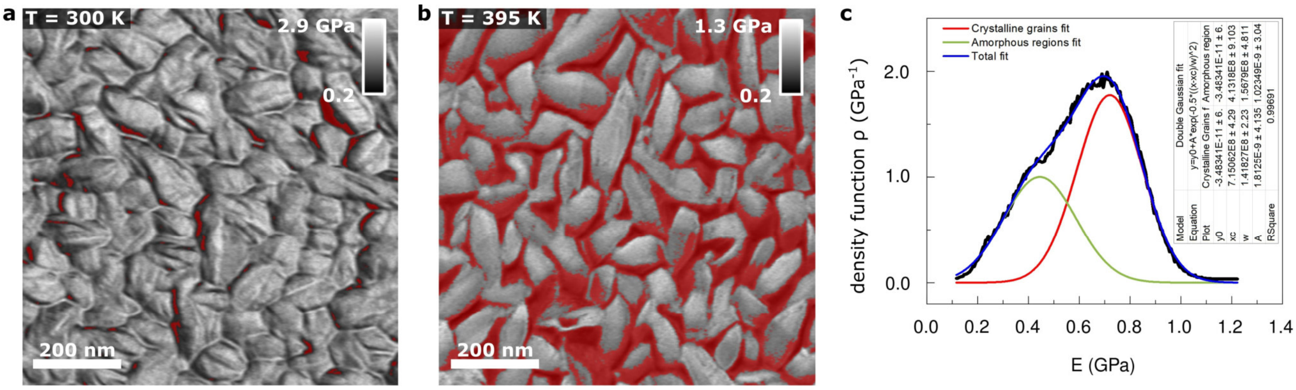

Supplementary Figure 21. Statistical analysis of the Young's modulus maps of P(VDF-TrFE). a, Young's modulus map after applying a threshold mask (sample at 300 K). **b,** Young's modulus map after applying a threshold mask (sample at 395 K). **c,** Young's modulus distribution at 395 K. The distributions are fitted with a double Gaussian function. Bimodal AM-FM data: A_{01} = 110 nm, A_1 = 74 nm, f_{01} = 56.454 kHz, k_1 = 1.75 N m^{-1}, Q_1 = 170.1, A_2 = 0.5 nm, f_{02} = 356427 kHz, k_2 = 69.76 N m^{-1}.

7. Dynamic mechanical analysis

In Supplementary Section 6, we conducted bimodal AFM measurements resulting in a high-resolution characterisation of the nanomechanical properties of P(VDF-TrFE). However, for semi-crystalline materials, such as these polymers, it is crucial to distinguish between micro- and macroscopic material properties. The macroscopic Young`s modulus of the bulk material of P(VDF-TrFE) can be measured using DMA measurements. For this purpose, we fabricated 10 µm thick free-standing polymer films according to Supplementary Section 2, detached from the substrate and then cut into appropriate sizes for the measurements. Supplementary Figure 22 shows a photo of a clamped P(VDF-TrFE) film in the DMA setup; we used the commercial TA Instruments DMA Q800. The temperature dependency of the macroscopic Young`s modulus of P(VDF-TrFE) is shown in Supplementary Figure 23a. At a temperature of 300 K, we measured a Young`s modulus of 2.1 GPa, which is in good agreement with the averaged value of 2 GPa obtained from bimodal AFM measurements. As the temperature increases, the Young`s modulus decreases linear. At the Curie temperature $T_C$ = 374 K, the Young`s modulus abruptly remains constant at a value of about 0.2 GPa (see Supplementary Figure 23a). The bimodal AFM measurements yield a Young`s modulus of 0.3 ± 0.1 GPa for the amorphous regions above T_C, which represent the dominant contribution to the macroscopic value determined with DMA. From bimodal AFM characterisation, we know that there still exist stiffer crystalline domains distributed inside the softer amorphous matrix. Macroscopically however, they are not measurable anymore, since individual crystalline domains are completely detached from each other. The amorphous regions form a network

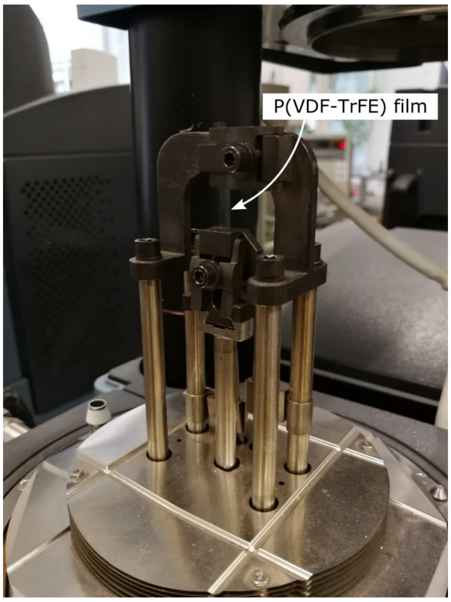

Supplementary Figure 22. DMA Setup. P(VDF-TrFE) film clamped in the DMA

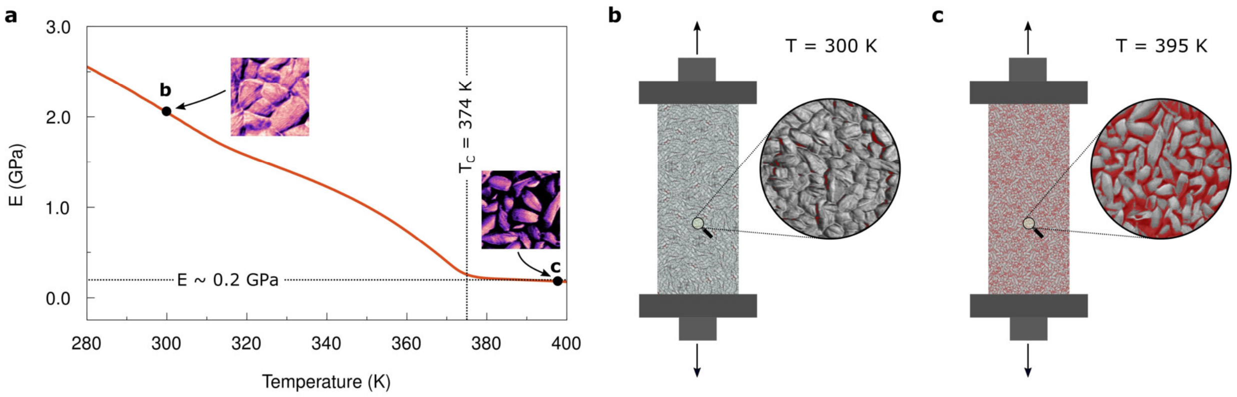

Supplementary Figure 23. Macroscopic Young's modulus of P(VDF-TrFE). a, Young's modulus of the bulk material of P(VDF-TrFE) as a function of temperature. **b, c,** Illustration for the change in the macroscopic Young's modulus above the Curie temperature T_C, resulting from a rearrangement of the amorphous regions. (grey: crystalline grains, red: amorphous regions).

around the crystalline grains, so that only the amorphous network is measurable macroscopically (see Supplementary Figure 23b,c). The fact that crystalline domains are embedded into a soft amorphous matrix has not only an impact on the mechanical properties of the polymer, but also the electromechanical properties are strongly influenced by this phenomenon.

8. *In situ* cantilever-based deflection measurements

8.1 Experimental section

Temperature dependent cantilever-based deflection measurements[52,53] on P(VDF-TrFE) capacitor structures (see Supplementary Section 2) were conducted to measure the induced strain under the application of an external electric field using a Sawyer-Tower configuration. Due to the thickness of the thin film capacitors, very small vertical displacements under influence of the driving signal (in the order of a few nm) needed to be recorded. As such, the highly sensitive optical lever readout, commonly implemented in commercial AFM, was seen as a suitable candidate to probe the bulk strain behaviour of the semi-crystalline P(VDF-TrFE) in the low frequency regime. For *in situ* cantilever-based deflection measurements, a Bruker Dimension Edge AFM was utilised. Commercial, Nanoworld PNP-TR AFM probes (k = 0.32 N/m) were used as the microcantilever probes. Probes with silicon nitride as tip and cantilever material were chosen to prevent the electrical interference caused by ground loops, compared to probes made of more conductive materials such as doped silicon. The setup used is shown as a block diagram in Supplementary Figure 24.

For the purpose of strain measurements at temperatures beyond the Curie temperature, the capacitor structure was mounted onto a highly stable temperature stage (see Supplementary Section 5). The vertical microcantilever deflection was recorded in the four-quadrant photodetector after laser beam reflection on the backside of the probe. The deflection was calibrated by recording the deflection signal against the movement of the z-axis positioning element in the scanning head. Once the tip enters hard contact with the sample surface, a linear response curve is obtained. The inverse of the slope is known as inverse optical lever sensitivity (InvOLS) and can be used to calibrate the photodetector deflection signal (proportional to a voltage) into vertical displacement in length units. This calibration is sensitive to the compliance of the sample surface that the AFM probe presses against, impacting the measured InvOLS. To avoid

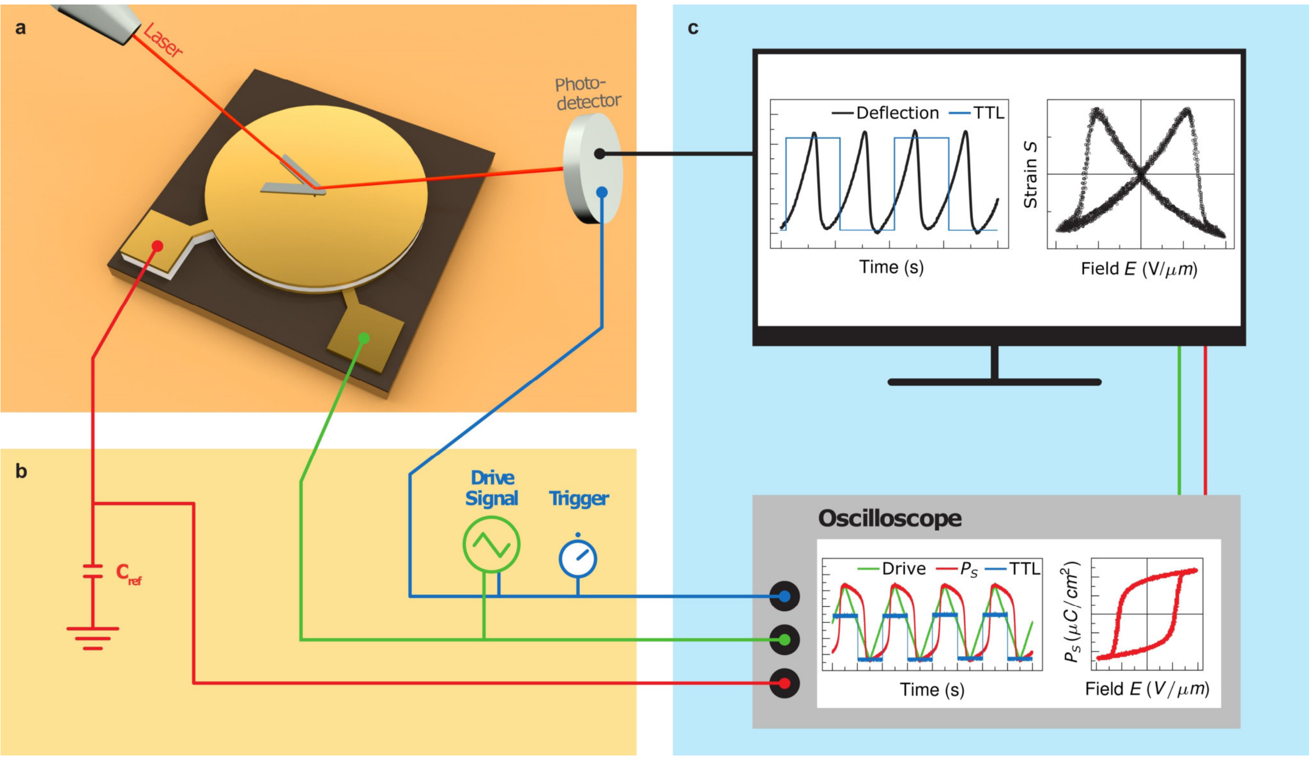

Supplementary Figure 24. Block diagram of *in situ* cantilever-based deflection measurements. a, AFM setup with a P(VDF-TrFE) capacitor. **b,** Triangular shaped driving signal induces ferroelectric polarisation switching in the capacitor, which can be measured via a Sawyer-Tower circuit. The microcantilever deflection and electrical measurements are synchronised in post-processing by feeding a TTL trigger signal from the waveform generator. **c,** Monitoring of the microcantilever deflection and the electric polarisation.

significant indentation effects, the silicon wafer surface surrounding the capacitor structure was used to land the probe for the calibration factor determination. After recording the InvOLS the probe was retracted and landed in contact mode on the top electrode of the capacitor. The probe was placed in an area close to the centre of the electrode to ensure that any substrate bending during electrical stimulation of the device was kept negligible, as further discussed in Supplementary Section 8.2. During time-dependent deflection acquisition when driving the device, the vertical scanner component was kept in open loop.

The device was connected in a Sawyer-Tower circuit and externally driven by application of a triangular waveform at a frequency of 10 Hz, enabling the simultaneous recording of the polarisation and induced

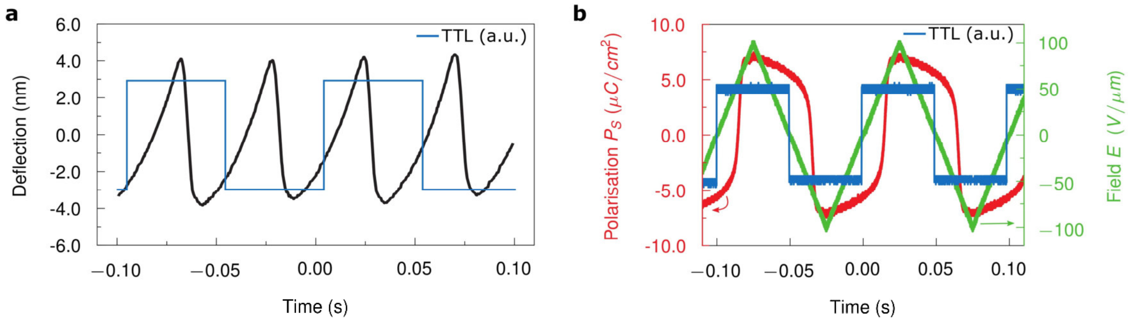

Supplementary Figure 25. *In situ* recorded signals of the bulk deformation of P(VDF-TrFE). a, AFM deflection data and TTL pulse (TTL) for signal phase matching. **b,** Electrical data acquired at the oscilloscope.

strain. To synchronise the mechanical displacement recorded in the AFM with the driving signal and voltage drop in the oscilloscope, a TTL trigger signal was provided as output by the waveform generator and fed into both instruments. Via post processing, it was possible to perform a synchronisation by phase matching the two square waveform signals recorded in the AFM controller and oscilloscope separately. The *in situ* recorded signals are shown in Supplementary Figure 25. Hysteresis and butterfly shaped strain vs. electric field curves were obtained by plotting the electrical displacement and induced strain against the driving voltage signal after converting into the appropriate units (see Supplementary Section 4).

8.2 Clamping effects

Piezoelectric measurements of thin films are commonly hindered by clamping the film onto a rigid substrate. Hence, it is crucial to estimate the contribution of clamping effects on the conducted measurements in this section. For instance, the in-plane strain of a fully clamped piezoelectric thin film induced by an electric field should be zero and, hence, the measured longitudinal piezoelectric constant

$$d_{33}^{\text{effective}} = d_{33}^{\text{intrinsic}} - \frac{2s_{13}^{f}}{s_{11}^{f}-s_{12}^{f}}\, d_{31}^{\text{intrinsic}} \qquad \text{(S15)}$$

is reduced with respect to the intrinsic $d_{33}^{\text{intrinsic}}$ of the material[54]. In equation (S15), s_{ij}^{f} are the elastic compliances of the piezoelectric thin film under constant electric field E and $d_{31}^{\text{intrinsic}}$ is the intrinsic

transverse piezoelectric coefficient. For P(VDF-TrFE), $d_{31}^{\text{intrinsic}}$ is positive. The elastic compliances s_{12} and s_{13} are negative, and s_{11} is positive but larger than s_{12}, consequently $d_{33}^{\text{effective}}$ gives an underestimation of $d_{33}^{\text{intrinsic}}$. In case of a fully clamped P(VDF-TrFE) film on a rigid substrate, the intrinsic piezoelectric constant would be reduced by about 30 %. We used the intrinsic piezoelectric constants $d_{31}^{\text{intrinsic}} = 10.7$ pm/V, $d_{33}^{\text{intrinsic}} = -33.5$ pm/V and the elastic compliances $s_{11} = 3.32 \cdot 10^{-10}\ \text{m}^2/\text{N}$, $s_{12} = -1.44 \cdot 10^{-10}\ \text{m}^2/\text{N}$, $s_{13} = -0.89 \cdot 10^{-10}\ \text{m}^2/\text{N}$ of P(VDF-TrFE) for the calculation[55]. We note that in equation (S15) the factor 2 in the second term originates from the assumption $d_{31}^{\text{intrinsic}} = d_{32}^{\text{intrinsic}}$, which is valid for P(VDF-TrFE) with $d_{32}^{\text{intrinsic}} = 10.1$ pm/V. However, this assumption is not correct for PVDF with $d_{31}^{\text{intrinsic}} = 28$ pm/V and $d_{32}^{\text{intrinsic}} = 4$ pm/V; here, we have to add both piezoelectric constants together ($d_{31}^{\text{intrinsic}} + d_{32}^{\text{intrinsic}}$) instead of $2d_{31}^{\text{intrinsic}}$ in the second term of equation (S15).

The model according to equation (S15) does not take into account clamping effects resulting from the electrodes, the aspect ratio of film thickness to lateral dimension of the capacitor or the aspect ratio of film thickness to the thickness of the electrodes. In a first approach, flexible bending of the substrate or clamped electrodes to the piezoelectric layer can be considered via

$$d_{33}^{\text{effective}} = d_{33}^{\text{intrinsic}} - \frac{2s_{13}^{f}}{s_{11}^{f} - s_{12}^{f}}\, d_{31}^{\text{intrinsic}} + \frac{2s_{13}^{s}}{s_{11}^{s} - s_{12}^{s}}\gamma\, d_{31}^{\text{intrinsic}}, \qquad \text{(S16)}$$

where $\gamma = G^f/G^s = [E^f/(1\text{-}\nu^f)]/\ [E^s/(1\text{-}\nu^s)]$ is the ratio of planar elastic modulus between the film and the substrate or electrode; here $E^{f/s}$ is the elastic modulus and $\nu^{f/s}$ is the Poisson ratio, respectively[56]. We see that equation (S16) is an extension of equation (S15), where the third term describes the elastic bending of the substrate or electrode. When a soft film is clamped to a stiff substrate or electrode, the third term vanishes and equation (S16) lead to equation (S15). The same is valid if the thickness of the film is much smaller than the thickness of the substrate, because the stress in the substrate gets negligible compared with that of the film. In our case, we can assume that the third term vanishes in that model, since

P(VDF-TrFE) is much softer than the stiff substrate; in addition, the film of P(VDF-TrFE) is much thinner than the substrate. To ensure that we do not have parasitic effects originating from elastic deformation and bending of the substrate, we measured the displacement not only on the capacitor, but also on the substrate. Along a line through the centre of the capacitor, we measured at various points the displacement (see Supplementary Figure 26a). The piezoelectric coefficients measured at these selected locations are shown in Supplementary Figure 26b. On the capacitor, the piezoelectric coefficients measured are constant within the measurement accuracy. Supplementary Figure 26c shows the butterfly curve at the centre of the capacitor with a piezoelectric coefficient of -26.2 pm/V. Almost no displacement is measurable on the substrate, i.e. elastic deformation or bending of the substrate can be neglected. Supplementary Figure 26d shows the displacement of the substrate during electrical stimulation of the piezoelectric thin film as a function of the applied field. The displacement of the substrate with less than 1 nm has a low impact compared to that of the piezoelectric thin film, which is more than 5 nm. Consequently, the substrate is rigid and additional elastic bending of that can be neglected in equation (S16). On the other hand, the top electrode with a thickness of 100 nm is much thinner than the piezoelectric film with a thickness of 1 μm. However, the elastic material properties differ and γ can be assumed to be small; consequently, the third term vanishes also for the top electrode. We note that equation (S16) does not consider any aspect ratios of the different layers of the capacitor-type test structures. Only a few analytical models are presented in literature, trying to consider the aspect ratios of the different layers[57]. The full set of design parameters are commonly studied via finite element analysis (FEA) simulations[56-60]. We realised FEA simulations using COMSOL. We implemented the exact dimensions of the capacitor and modelled an applied DC voltage to the electrodes between the piezoelectric P(VDF-TrFE) with an intrinsic piezoelectric coefficient of -33.5 pm/V, resulting in a displacement of the capacitor depending on the applied voltage. For instance, Supplementary Figure 27a shows the simulated displacement of the capacitor for -10 V. Measuring the displacement of the capacitor as a function of the applied voltage

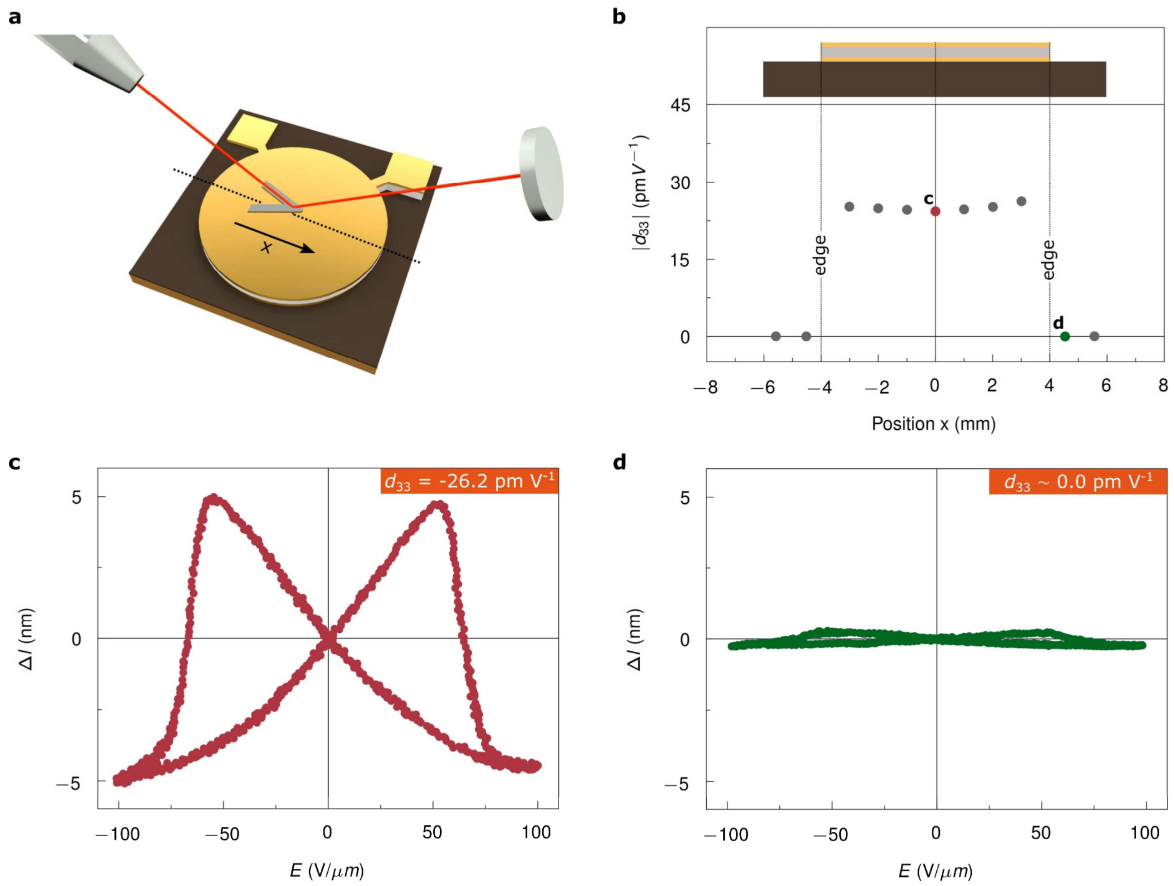

Supplementary Figure 26. Analysis of substrate bending during piezoelectric measurements. a, Schematic illustration, showing the measurement procedure along a line through the centre of the capacitor. **b,** Measured piezoelectric coefficient at different points of the capacitor and the substrate. **c,** Displacement at the centre of the capacitor (butterfly curve). **d,** Displacement on the substrate next to the capacitor.

results in a linear relation. The slope is the value of the measured piezoelectric coefficient $d_{33}^{\mathrm{effective}}$. From the FEA simulations, we can estimate the impact of clamping effects on our piezoelectric measurements (see Supplementary Figure 27b). The measured piezoelectric coefficient of -26.2 pm/V in Supplementary Section 4 originates from an intrinsic piezoelectric coefficient of around -35.4 pm/V. Both values are in fair agreement with those reported in literature for bulk, unclamped P(VDF-TrFE) thin films with values ranging from -25 pm/V to -40 pm/V[19-21]. We note that the results of the FEA simulations are in good agreement with the analytical results expected according to the equations (S15) and (S16).

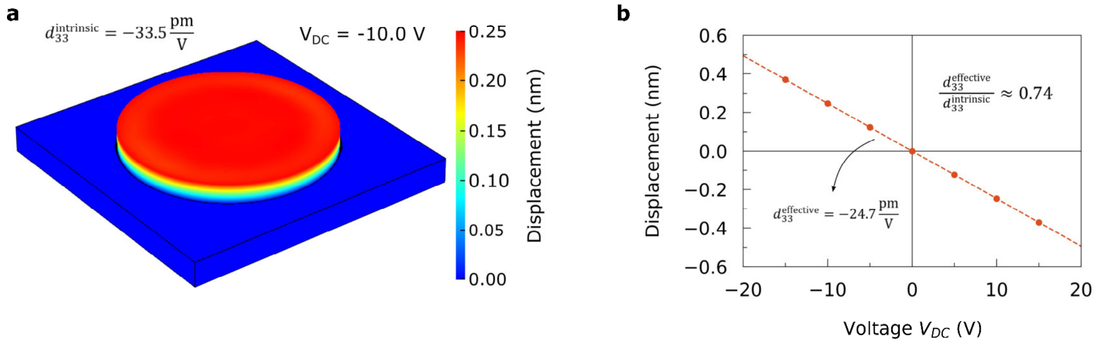

Supplementary Figure 27. Analysis of clamping effects using FEA simulations. a, Simulated displacement of the capacitor for a DC voltage of -10 V. **b,** Extracted displacement from the FEA simulation in the centre of the capacitor as a function of applied DC voltage. The slope of the linear relation yields the measured piezoelectric coefficient.

In the high-temperature paraelectric phase of P(VDF-TrFE), the morphology and mechanical properties differ from the low-temperature ferroelectric phase. Since the mechanical properties change, one should consider that clamping effects might prevent any electromechanical response of the polymer film. In the high-temperature paraelectric phase, we measure in the centre of the capacitor-type test structure only a tiny electrostrain of less than 0.07 % for an applied field of 100 V/µm (see Supplementary Section 9). The macroscopic response is infinitesimal compared to the microscopic electrostrain of around 1 % originating from the crystalline domains. The crystalline domains are embedded into a soft amorphous matrix (see Supplementary Section 6). Internal losses between the crystalline domains and the surrounding amorphous matrix prevents a propagation of the microscopic electrostrain to the macroscopic level. Additionally, clamping effects diminish macroscopic deformations of the polymer film. A detailed analysis of the clamping effects is unfeasible and not trustful without the knowledge of the elastic stiffness matrix of the composite structure of P(VDF-TrFE) in the high-temperature paraelectric phase. However, under the same clamping conditions of the polymer film, the crystalline domains inside of the amorphous matrix exhibit a strong electromechanical response, which corroborates the statement that the amorphous matrix counterbalances the electrostrain towards the macroscopic level. Without the outer amorphous matrix the

electrostrain would be measurable macroscopically. But also if clamping effects prevent a macroscopic response, we can argue that the compensation of the microscopic response arises from the surrounding amorphous matrix. Only through microscopic measurements, such as *in situ* XRD measurements, one can reveal this microscopic electrostrain. The mechanism and the dynamic behaviour of the amorphous matrix and the interaction between both material phases are interesting fields to study in future works.

9. *In situ* X-ray diffraction measurements

9.1 Experimental section

In Supplementary Section 8, we measured the macroscopic strain of the ferroelectric P(VDF-TrFE). Since the polymer is semi-crystalline, we conducted *in situ* grazing incidence XRD measurements to distinguish the macro- and microscopic strain behaviour of the polymer. Both measurements were performed below (T = 300 K) and above (T = 395 K) the Curie transition (T_C = 374 K) using the heating setup described in Supplementary Section 5. Supplementary Figure 28 shows the XRD patterns at these two temperatures. Below the Curie transition in the low-temperature ferroelectric state, we measure the characteristic XRD peak at 2θ = 19.7° for P(VDF-TrFE). Above the Curie transition in the high-temperature paraelectric state, we measure an XRD peak at 2θ = 17.8° corresponding to the high-temperature paraelectric phase.

In situ analyses of the shift of the XRD patterns as a function of the applied electric field in the ferro- and paraelectric state of P(VDF-TrFE) were performed. For that purpose, we applied a DC voltage to the capacitors and measured the corresponding out-of-plane scattering between angles of 2θ = 16.0° and 2θ = 24.0° in the specular plane. Repeating this procedure for different voltages, we get the lattice

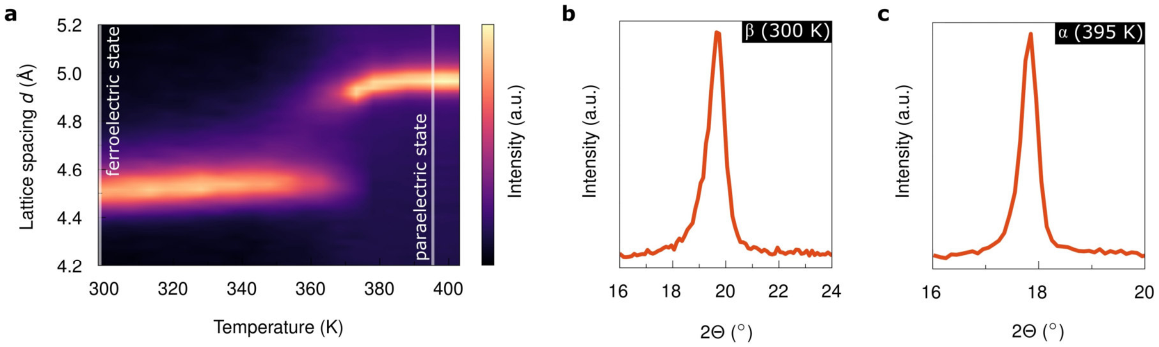

Supplementary Figure 28. XRD patterns of P(VDF-TrFE) below and above the Curie transition. a, Crystalline phase transition from the ferro- to paraelectric state. **b,** XRD pattern of the ferroelectric state at a temperature of 300 K. **c,** XRD pattern of the paraelectric state at a temperature of 395 K. The ratio VDF:TrFE is 70:30 mol%.

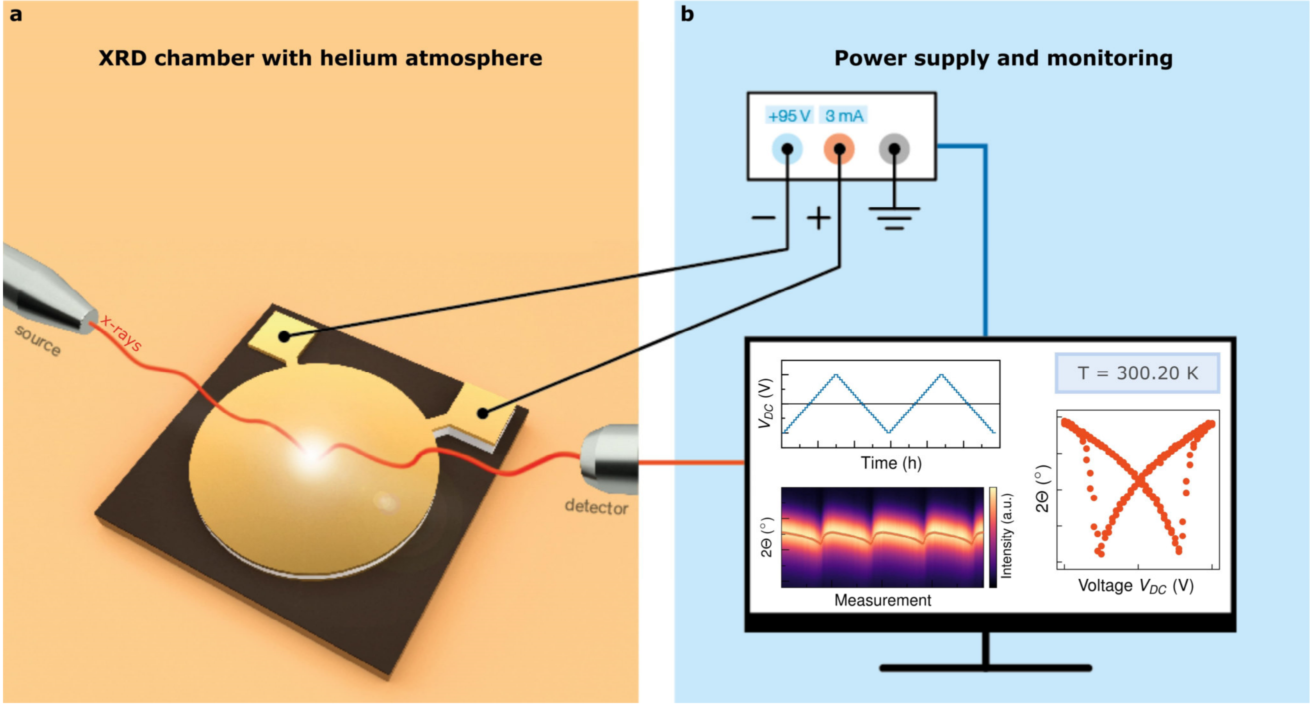

Supplementary Figure 29. Setup for the *in situ* XRD measurements. a, XRD chamber with helium atmosphere and the electrical connected capacitors. **b,** Power supply and monitoring of the measurement parameters.

constant of the crystalline domains as a function of the applied voltage. The used measurement setup is illustrated as a simplified block diagram in Supplementary Figure 29. The XRD diffractometer used for our experiments was an X'Pert Pro PANalytical. The X-ray beam was focused at the centre of the sample by a mirror. The incidence angle on the sample was set to 5.0°, matching the XRD footprint to the active area of the capacitors. We tested this by measuring the XRD signal between angles of $2\theta = 5.0°$ and $2\theta = 90.0°$. The scattering collected not only shows the characteristic signals of P(VDF-TrFE), but also of Au, Cr, Si and SiO_2, which verifies that the X-ray beam passed through all layers of the capacitor structure, including the entire volume of the P(VDF-TrFE) thin film. In order to improve the signal-to-noise ratio, all *in situ* XRD measurements were conducted under helium atmosphere.

The *in situ* XRD measurements were performed quasi-statically, meaning that the applied DC voltage was stepwise increased/decreased and the X-ray scattering was measured during time intervals of constant

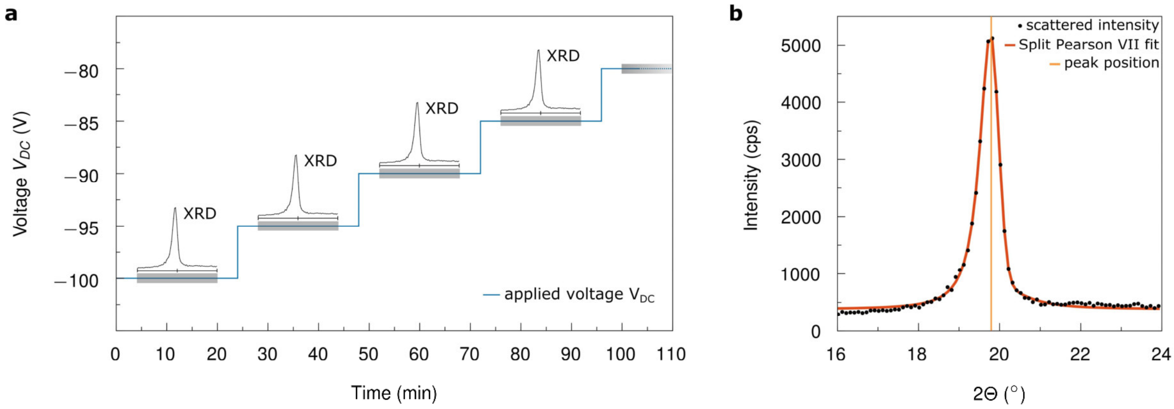

Supplementary Figure 30. Measurement protocol for the *in situ* XRD measurements. a, Time course of the applied voltage and the conducted *in situ* XRD measurements. **b,** The extracted (110)/(200) diffraction peak of the low-temperature β-phase of P(VDF-TrFE) for an applied voltage of -100 V, respectively. The red line is a Split Pearson VII fit to scattered intensities.

voltage. The time interval for constant voltage was set to 24 min; in between, we measured the XRD pattern for 16 min (see Supplementary Figure 30a). The time between measurement and change of the applied voltage was 4 min, ensuring that mismatches between the applied voltages and the corresponding XRD patterns did not occur over the entire time of the measurement. In addition, sufficient time was given to the ferroelectric to react to the change of the applied voltage. The applied voltage ranged from -100 V to +100 V. We measured two cycles, i.e. the entire measurement run took about 65 hours. As described in Supplementary Section 5, the temperature was kept constant and checked by means of two thermocouples. Before each measurement, the temperature was allowed to reach steady-state over a time of around 30 min. Subsequently, the voltage was ramped to -100 V in 10 V steps within 10 min before the first measurement.

For the evaluation of the data, a Split Pearson VII function was fitted to each scattered intensity characteristics to extract the peak positions. A typical fit of the low-temperature Bragg peak, while applying a voltage of -100 V to the capacitor, is shown in Supplementary Figure 30b. The standard deviation of the fit was on average at a value of about 0.005°, which corresponds to an uncertainty in the lattice spacing of about 0.001 Å.

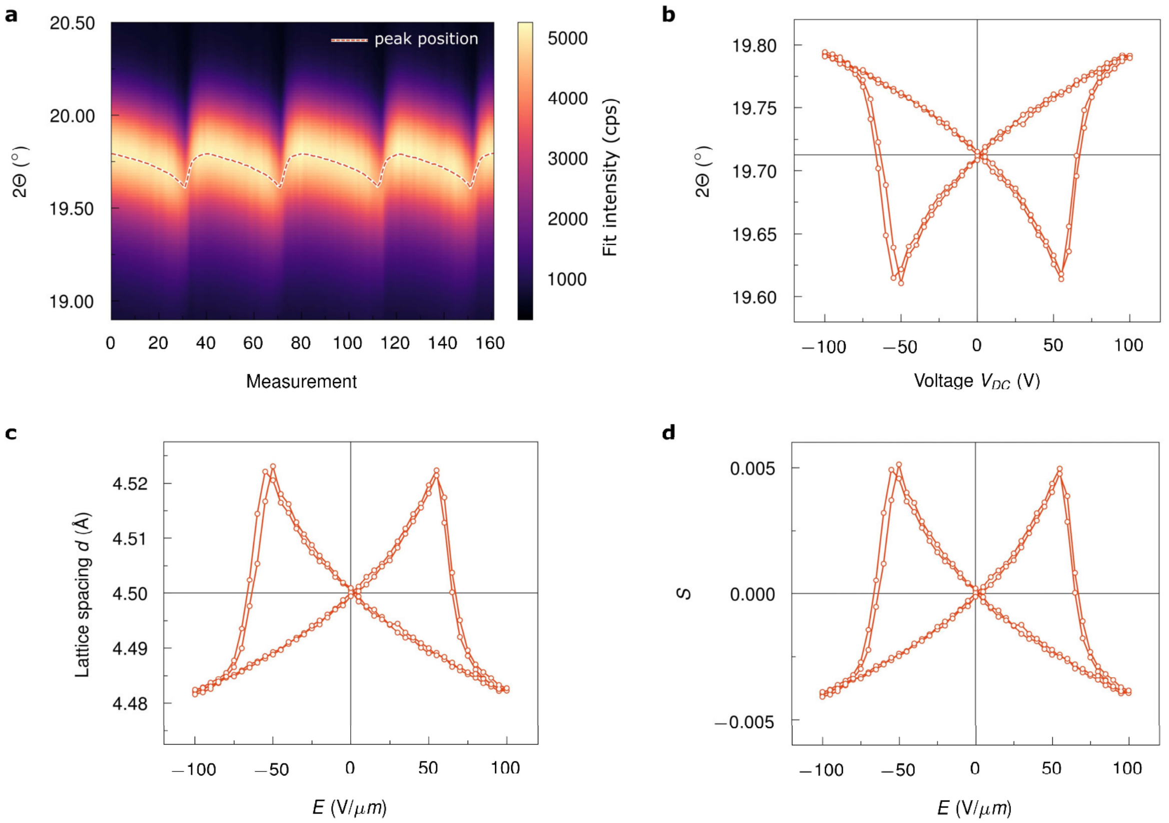

Supplementary Figure 31. Microscopic electromechanical displacement of P(VDF-TrFE) at 300 K. a, XRD scattered intensities (represented as Split Pearson VII fit) and the corresponding peak position, showing the response of the lattice to an external voltage. **b,** Angle 2θ of the peak position as a function of the applied voltage. **c, d,** Extracted lattice spacing *d* and strain *S* as a function of the electric field.

Hence, the position can be determined as a function of the applied voltage. Supplementary Figure 30a shows the fitted scattered intensities and the resulting peak position for each individual measurement. The resulting butterfly curves are shown in Supplementary Figure 31. We determined the lattice spacing using Bragg`s law and plotted the microstrain S as the relative change in lattice spacing, $S = \Delta l/l_0$, where l_0 is the value at zero electric field. The external electric field E was calculated from the applied voltage and the layer thickness. Using the model presented in Supplementary Section 4.1, we extracted a longitudinal electrostriction coefficient of around Q_{33} = -1.9 m^4/C^2 and a piezoelectric coefficient of d_{33} = -29.3 pm/V from the microscopic strain behaviour of P(VDF-TrFE) (see Supplementary Figure 31d).

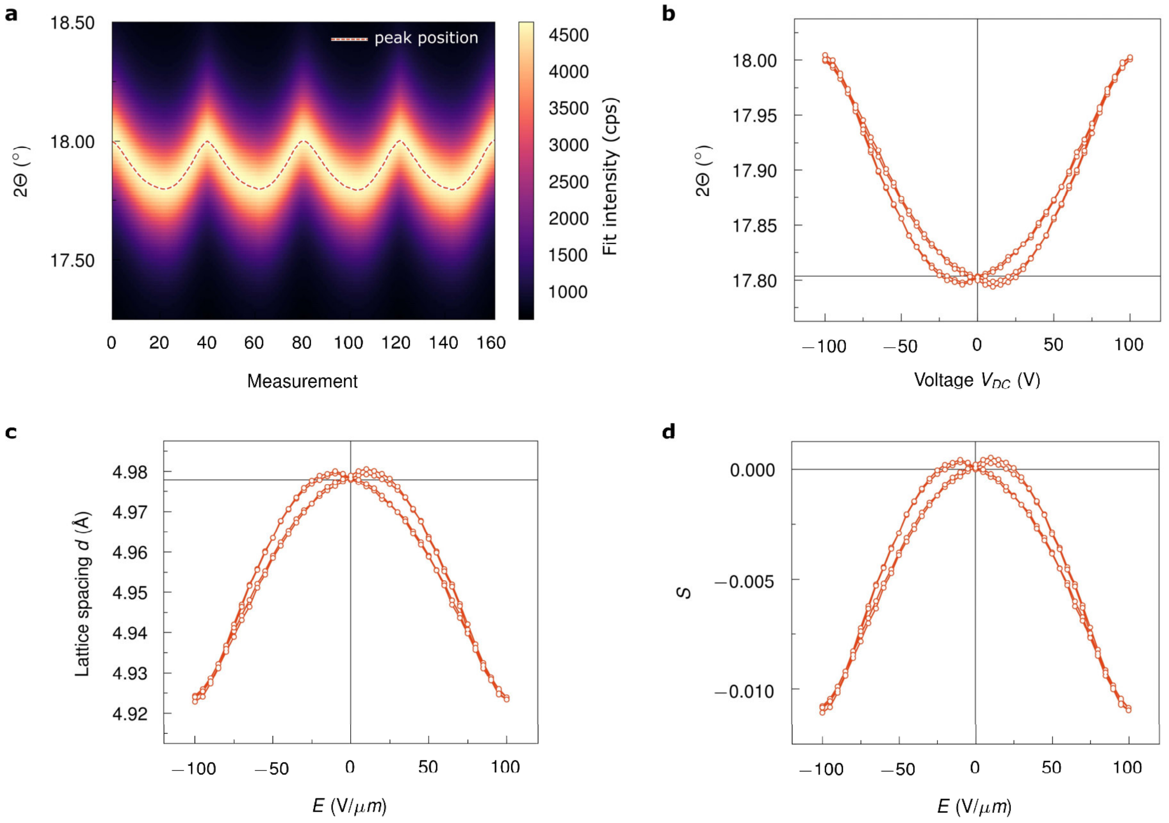

Supplementary Figure 32. Microscopic electromechanical displacement of P(VDF-TrFE) at 395 K. a, XRD scattered intensities (represented as Split Pearson VII fit) and the corresponding peak position, showing the response of the lattice to an external voltage. **b,** Angle 2θ of the peak position as a function of the applied voltage. **c, d,** Extracted lattice spacing *d* and strain *S* as a function of the electric field.

The results for the *in situ* XRD measurements in the high-temperature state at 395 K are shown in Supplementary Figure 32. Although the material is in the paraelectric phase, a strain of the crystalline domains of more than 1% is measured. The electromechanical response is strongly electrostrictive with an electrostriction coefficient of around Q_{33} = -10.2 m^4/C^2. However, there is still a hysteretic response observable, resulting from field-induced polar nanoregions (see Supplementary Section 4). We did also a field-dependent study on the electromechanical response of the crystalline domains above the Curie transition (see Supplementary Section 4.3). It was found that upon a sufficient electric field of around 80 V/μm a bistability in the electrostrain characteristic occurs. However, this strong electromechanical

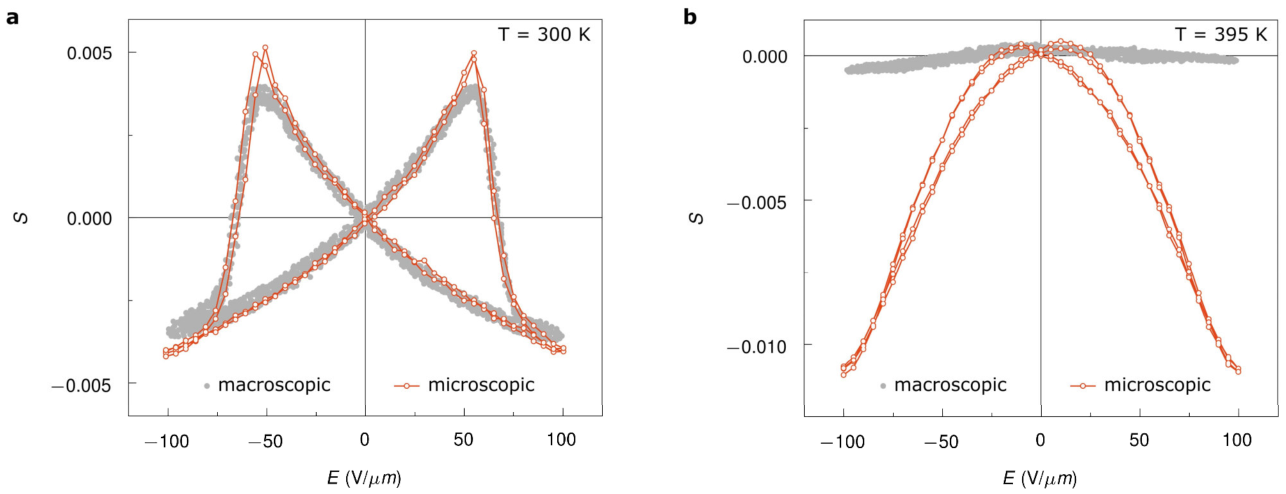

Supplementary Figure 33. Comparison of macro- and microscopic strain behaviour of P(VDF-TrFE). a, Strain as a function of electric field in the low-temperature ferroelectric state. **b,** Strain as a function of electric field in the high-temperature paraelectric state.

response of the crystalline domains is not observable macroscopically on the bulk material, since they are embedded into a soft amorphous matrix (see Supplementary Section 6). The amorphous region absorbs the larger electromechanical displacement by the crystalline domains. For a direct comparison, the macro- and microscopic measurements are shown in Supplementary Figure 33.

We note that we used the same samples for the macro- and microscopic strain measurements in order to compare the results. In addition, before conducting *in situ* XRD measurements, we tested the stability of the peak position for 250 measurements for over 65 hours. A statistical analysis showed that the shift of the Bragg peaks with an accuracy of about ± 0.001 Å.

9.2 Clamping effects

In Supplementary Section 8.2, we discussed the influence of the substrate clamping on the macroscopic lateral displacement of the ferroelectric thin film. Using *in situ* XRD to measure the electromechanical response of the thin film is almost unaffected by clamping effects[19,20]. Thus, the extracted piezoelectric

coefficient with XRD is higher than the value measured with the microcantilever deflection measurements (see Supplementary Section 8 and 9). However, we analysed potential clamping effects during the *in situ* XRD measurements in more detail.

A possible measurement artefact could be induced by clamping of the portion of crystals in close proximity to the substrate or the top electrode. As a consequence, their displacement would be hindered leading to a field-dependent broadening of the measured diffraction peak[19]. To check, if there is any broadening, we observed the full width at half maximum (FWHM) of the diffraction peak. Supplementary Figure 34 shows the FWHM as a function of the applied field for *in situ* XRD measurements at a temperature of 300 K and 395 K. At 300 K, the width is broader only during polarisation switching around the coercive voltage. Since the FWHM shows elsewhere no obvious variations elsewhere, we can conclude that clamping effects are weak. At 395 K, the width of the diffraction peak shows no change during the measurements, meaning that the change in crystal lattice is completely free from external influences like clamping effects. This is attributed to the fact that the crystals are suspended by an amorphous matrix. As a final remark, it should be noted that the Curie transition occurs without any loss in orientation character[36]. However, it would be interesting to analyse the crystal orientation in future works as done in the literature[61-63].

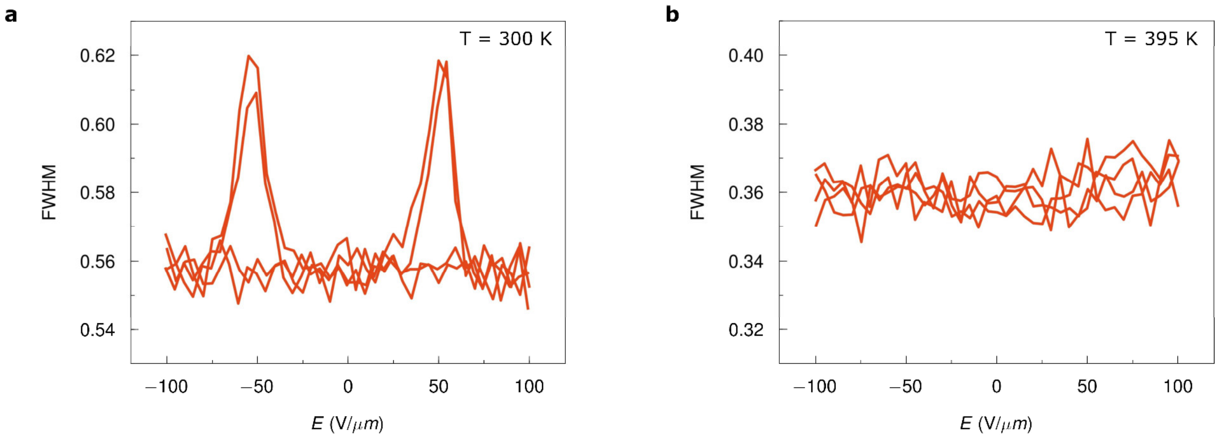

Supplementary Figure 34. Measured FWHM as a function of applied field. a, FWHM during the *in situ* XRD measurements at the low-temperature state at 300 K. **b,** FWHM during the *in situ* XRD measurements at the high-temperature state at 395 K.

References

1. Lovinger, A. J. Ferroelectric polymers. *Science* **220,** 1115-1121 (1983).
2. Tashiro, K. & Tanaka, R. Structural correlation between crystal lattice and lamellar morphology in the ferroelectric phase transition of vinylidene fluoride-trifluoroethylene copolymers as revealed by the simultaneous measurements of wide-angle and small-angle X-ray scatterings. *Polymer* **47,** 5433-5444 (2006).
3. García-Gutíerrez, et al. Understanding crystallization features of P(VDF-TrFE) copolymers under confinement to optimize ferroelectricity in nanostructures. *Nanoscale* **5,** 6006 (2013).
4. Martin, J. et al. Relaxations and relaxor-ferroelectric-like response of nanotubularly confined poly(vinylidene fluoride). *Chem. Mat.* **29,** 3515-3525 (2017).
5. Meng, N., et al. Multiscale understanding of electric polarization in poly(vinylidene fluoride)-based ferroelectric polymers. *J. Mater. Chem. C* (2020).
6. Zhang, Q. M., Bharti, V. & Kavarnos, G. Poly(vinylidene fluoride) (PVDF) and its copolymers. *Encyclopedia of Smart Materials* edited by M. Schwartz, 807-825 (2002).
7. Tashiro, K. Crystal structure and phase transition of PVDF and related copolymers. In Ferroelectric Polymers: Chemsitry, Physics, and Applications; Nalwa, H. S., Ed.; Marcel Dekker: New York; Chapter 2, 63-182 (1995)
8. Qian, J., et al. Unveiling the piezoelectric nature of polar α-phase P(VDF-TrFE) at quasi-two-dimensional limit. *Sci. Rep.* **8** (2018).
9. Li, M., et al. Revisiting the δ-phase of poly(vinylidene fluoride) for solution-processed ferroelectric thin films. *Nat. Mater.* **12,** 433-438 (2013).
10. Martin, J. et al. Solid-state-processing of δ-PVDF. *Mater. Horiz.* **4,** 408-414 (2017).

11. Chen, S., Yao, K., Tay, F. E. H. & Chew, L. L. S. Comparative investigation of the structure and properties of ferroelectric poly(vinylidene fluoride) and poly(vinylidene fluoride-trifluoroethylene) thin films crystallized on substrates. *J. Appl. Polym. Sci.* **116,** 3331-3337 (2010).
12. Chung, T. C. & Petchsuk, A. Synthesis and properties of ferroelectric fluoroterpolymers with Curie transition at ambient temperature. *Macromolecules* **35,** 7678-7684 (2002).
13. Tashiro, K. & Hama, H. Structural changes in isothermal crystallization processes of synthetic polymers studied by time-resolved measurements of synchrotron-sourced X-ray scatterings and vibrational spectra. *Macromol. Res.* **12,** 1-10 (2004).
14. Zhang, Q. M., Bharti, V. & Zhao, X. Giant electrostriction and relaxor ferroelectric behavior in electron-irridiated poly(vinylidene fluoride-trifluoroethylene) copolymer. *Science* **280,** 2101-2104 (1998).
15. Su, R. et al. Ferroelectric behavior in the high temperature paraelectric phase in a poly(vinylidene fluoride-*co*-trifluoroethylene) random copolymer. *Polymer* **53,** 728-739 (2012).
16. Yang, L., et al. Novel polymer ferroelectric behavior via crystal isomorphism and the nanoconfinement effect. *Polymer* **54,** 1709-1728 (2013).
17. Miller, S. L., et al. Device modeling of ferroelectric capacitors. *J. Appl. Phys.* **68,** 6463-6471 (1990).
18. Lue, H. T., Wu, C. J. & Tseng, T. Y. Device modeling of ferroelectric memory field-effect transistor (FeMFET). *IEEE Trans. Electron Devices* **49,** 1790-1798 (2002).
19. Katsouras, I., et al. The negative piezoelectric effect of the ferroelectric polymer poly(vinylidene fluoride). *Nat. Mater.* **15,** 78-84 (2016).
20. You, L., et al. Origin of giant negative piezoelectricity in a layered van der waals ferroelectric. *Sci. Adv.* **5,** 3780 (2019).

21. Furukawa, T. & Seo, N. Electrostriction as the origin of piezoelectricity in ferroelectric polymers. *Jpn. J. Appl. Phys.* **29,** 675–680 (1990).
22. Mai, M, Leschhorn, A. & Kliem, H. The field and temperature dependence of hysteresis loops in P(VDF-TrFE) copolymer films. *Physica B* **456,** 306-311 (2015).
23. Furukawa, T. Ferroelectric properties of vinylidene fluoride copolymers. *Phase Transit.* **18,** 143-211 (1989).
24. Li, M. et al. Ferroelectric phase diagram of PVDF:PMMA. *Macromolecules* **45,** 7477-7485 (2012).
25. Güthner, P., Ritter, T. & Dransfeld, K. Temperature dependence of the piezoelectric constant of thin PVDF and P(VDF-TrFE) films. Ferroelectrics **127**, 7-11 (1992).
26. Hafner, J., Teuschel, M., Schneider, M. & Schmid, U. Origin of the strong temperature effect on the piezoelectric response of the ferroelectric (co-)polymer P(VDF_{70}-$TrFE_{30}$). *Polymer* **170**, 1-6 (2019).
27. Cowley, R. A., et al. Relaxing with relaxors: a review of relaxor ferroelectrics. *Adv. Phys.* **60,** 229-327 (2011).
28. Pramanick, A., et al. Origin of dielectric relaxor behavior in PVDF-based copolymer and terpolymer films. *AIP Adv.* **8,** 045204 (2018).
29. Pramanick, A., et al. Ferroelectric to paraelectric phase transition mechanism in poled PVDF-TrFE copolymer films. *Phys. Rev. B* **96,** 174103 (2017).
30. Arifin, D. E. S. & Ruan, J. J. Study on the Curie transition of P(VDF-TrFE) copolymer. *Mater. Sci. Eng.* **299,** 012056 (2017).
31. Furukawa, T. & Wen, J. X. Electrostriction and piezoelectricity in ferroelectric polymers. *Jpn. J. Appl. Phys.* **23,** 677–679 (1984).

32. Green, S. J., Rabe, J. P. & Rabolt, J. F. Studies of chain conformation above the Curie point in a vinylidene fluoride/trifluoroethylene random copolymer. *Macromolecules* **19,** 1725-1728 (1986).
33. Kim, J. K., Reynolds, N. M. & Hsu, S. L. Spectroscopic analysis of the crystalline and amorphous phases in a vinylidene fluoride/trifluoroethylene copolymer. *Macromolecules* **22,** 4395-4401 (1989).
34. Zaitsu, K., Lee, S., Ishibe, K., Sekitani, T. & Someya, T. A field-cycle-induced high-dielectric phase in ferroelectric copolymer. *J. Appl. Phys.* **107,** 114506 (2010).
35. Tashiro, K., Kaito, H. & Kobayashi, M. Structural changes in ferroelectric phase transitions of vinylidene fluoride-tetrafluoroethylene copolymers: 1. Vinylidene fluoride content dependence of the transition behaviour. *Polymer* **33,** 2915-2928 (1992).
36. Tashiro, K. & Kobayashi, M. Vibrational spectroscopic study of the ferroelectric phase transition in vinylidene fluoride-trifluoroethylene copolymers: 1. Temperature dependence of the Raman spectra. *Polymer* **29,** 426-436 (1988).
37. Tashiro, K., Takano, K., Kobayashi, M., Chatani, Y. & Tadokoro, H. Structure and ferroelectric phase transition of vinylidene fluoride-trifluoroethylene copolymers: 2. VDF 55% copolymer. *Polymer* **25,** 195-208 (1984).
38. Tashiro, K., Nakamura, M., Kobayashi, M., Chatani, Y. & Tadokoro, H. Polarization inversion current and ferroelectric phase transition of vinylidene fluoride-trifluoroethylene copolymer. *Macromolecules* **17,** 1452-1455 (1984).
39. Reynolds, N. M., Kim, J. K., Chang, C. & Hsu, S. L. Spectroscopic analysis of the electric field induced structural changes in vinylidene fluoride/trifluoroethylene copolymers. *Macromolecules* **22,** 1092-1100 (1989).

40. Jin Kim, K., Reynolds, N. M. & Hsu, S. L. Spectroscopic studies on the effect of field strength upon the Curie transition of a VDF/TrFE copolymer. J. Polym. Sci., Part B: Polym. Phys. **31,** 1555−1566 (1993).
41. Lee, J. S., Prabu, A. A., Kim, J. K. & Cheolmin, P. The effect of an external electric field on solid-state phase transition of P(VDF/TrFE)(72/28). *Fibers Polym.* **8,** 456-462 (2007).
42. Poulsen, M., Sorokin, A. V., Adenwalla, S., Ducharme, S. & Fridkin, V. M. Effects of an external electric field on the ferroelectric-paraelectric phase transition in polyvinylidene fluoride-trifluoroethylene copolymer Langmuir-Blodgett films. *J. Appl. Phys.* **103,** 034116 (2008).
43. Mehner, E., et al. Anomalous ferroelectricity in P(VDF_{70}-$TrFE_{30}$). *Ferroelectrics* **510,** 132-151 (2017).
44. Davis, G. T., McKinney, J. E., Broadhurst, M. G. & Roth, S. C. Electric-field-induced phase changes in poly(vinylidene fluoride). *J. Appl. Phys.* **49,** 4998 (1978).
45. Broekmaat, J., Brinkman, A., Blank, D. H. A. & Rijnders, G. High temperature surface imaging using atomic force microscopy. *Appl. Phys. Lett.* **92,** 043102 (2008).
46. VanLandinghama, M., et al. Characterization of nanoscale property variations in polymer composite systems. *Compos. Part A: Appl. S.* **30,** 75-83 (1999).
47. Garcia, R. & Herruzo, E. T. The emergence of multifrequency force microscopy. *Nat. Nanotechnol.* **7,** 217–226 (2012).
48. Benaglia, S., et al. Fast and high-resolution mapping of elastic properties of biomolecules and polymers with bimodal AFM. *Nat. Protoc.* **13,** 2890–2907 (2018).
49. Amo, C.A., Perrino, A. P., Payam, A. F. & Garcia, R. Mapping Elastic Properties of Heterogeneous Materials in Liquid with Angstrom-Scale Resolution. *ACS Nano* **11,** 8650–8659 (2017).

50. Benaglia, S., Amo, C. A., Garcia, R. Fast, quantitative and high resolution mapping of viscoelastic properties with bimodal AFM. *Nanoscale* **11**, 15289-15297 (2019).
51. Labuda, A. et al. Calibration of higher eigenmodes of cantilevers. *Rev. Sci. Instrum.* **87,** 073705 (2016).
52. Christman, J. A., Woolcott, R. R., Kingon, A. I. & Nemanich, R.J. Piezoelectric measurements with atomic force microscopy. *Appl. Phys. Lett.* **73,** 3851 (1998).
53. Toprak, A. & Tigli, O. Comprehensive characterization of PVDF-TrFE thin films for microeletromechanical system applications. *J. Mater. Sci.: Mater. Electron.* **28,** 15877-15885 (2017).
54. Lefki, K. & Dormans, J. M. Measurement of piezoelectric coefficients of ferroelectric thin films. *J. Appl. Phys.* **76,** 1764-1767 (1994).
55. Safari, A. & Akdogan, E. K. Piezoelectric and acoustic materials for transducer applications. (Springer Science & Business Media, 2008)
56. Chen, L., Li, J. H., Slutsker, J., Ouyang, J. & Roytburd, A. L. Contribution of substrate to converse piezoelectric response of constrained thin films. *J. Mater. Res.* **19,** 2853 (2004).
57. Wang, J. H. Top electrode size effects in the piezoresponse force microscopy of piezoelectric thin films attached to a rigid substrate. *Smart Mater. Struct.* **26,** 105045 (2017).
58. Wang, Z., Lau, G. K., Zhu, W. & Chao, C. Influence of test capacitor features on piezoelectric and dielectric measurement of ferroelectric films. *IEEE Trans. Ultrason. Ferroelectr. Frequ. Control* **53,** 15 (2006).
59. Leighton, G. J. T. & Huang, Z. Accurate measurement of the piezoelectric coefficient of thin films by eliminating the substrate bending effect using spatial scanning laser vibrometry. *Smart Mater. Struct.* **19,** 065011 (2010).

60. Wang, H., Zhang, Q. M., Cross, L. E. & Sykes, A. O. Clamping effect on the piezoelectric properties of poly(vinylidene fluoride-trifluoroethylene) copolymer. *Ferroelectrics* **150,** 255-266 (2017).
61. Park, Y. J., et al. Molecular and crystalline microstructure of ferroelectric poly(vinylidene fluoride-*co*-trifluoroethylene) ultrathin films on bare and self-assembled monolayer-modified Au substrates. *Macromolecules* **41,** 109-119 (2008).
62. Jung, H. J., et al. Shear-induced ordering of ferroelectric crystals in spin-coated thin poly(vinylidene fluoride-co-trifluoroethylene) films. *Macromolecules* **42,** 4148-4154 (2009).
63. Andrew, C., et al. Polarization mechanisms in P(VDF-TrFE) ferroelectric thin films. *Phys. Status Solidi RRL* 1800340 (2018).
